# Goals for Adherence with Low-cost Incentives (GOALS): a protocol for a randomized controlled trial evaluating the impact of small airtime incentives on ART adherence among young people living with HIV in Kampala, Uganda

**DOI:** 10.1186/s13063-023-07449-z

**Published:** 2023-08-09

**Authors:** Sebastian Linnemayr, Haijing Crystal Huang, Zachary Wagner, Faith Kemunto Onkundi, Barbara Mukasa, Mary Odiit

**Affiliations:** 1 RAND Corporation, Santa Monica, USA; 2 IDinsight, San Francisco, USA; 3 Mildmay Uganda, Kampala, Uganda

**Keywords:** HIV/AIDS, Behavioral Economics, Incentives, Goals, Uganda, Global Health, Randomized controlled trial

## Abstract

**Background:**

Treatment outcomes of HIV-positive individuals are threatened by low antiretroviral therapy (ART) adherence, a problem that is particularly acute among youth. Incentives are a promising tool to support ART adherence, but traditional incentive designs rewarding uniformly high levels of the desired health behavior may demotivate those with low levels of the behavior. In this study, we investigate the effectiveness of alternative approaches to target-setting for incentive eligibility using subgoals (i.e., individual-specific, interim targets leading up to the optimal target).

**Methods / design:**

We will enroll 628 HIV-positive youth between ages 15 and 30 into a 3-year randomized controlled trial. Participants will be randomized 1:1:1:1 to a control arm or one of three intervention arms (*n* = 157 each) that allow them to enter a prize drawing for small incentives if their ART adherence meets the given goal. In the first arm (T1, assigned subgoal), goals will be externally assigned and adapted to their initial adherence level. In the second arm (T2, participatory subgoal), participants can set their own interim goals. In the third arm (T3, fixed goal), all participants must reach the same target goal of 90% adherence. T1 and T2 participants are required to reach 90% adherence by month 12 to participate in a larger prize drawing. The control group receives the usual standard of care. All four groups will receive weekly motivational messages; the three treatment groups will additionally receive reminders of their upcoming prize drawing. Adherence will be measured continuously throughout the intervention period using electronic devices and for 12 months post-intervention. Surveys will be conducted at baseline and every 6 months. Viral loads will be measured annually. The primary outcome is Wisepill-measured adherence and a binary measure for whether the person took at least 90% of their pills. The secondary outcome is the log-transformed viral load as a continuous measure.

**Discussion:**

Our study is one of the first to apply insights about the psychology and behavioral economics of goal-setting to the design of incentives, by testing whether conditioning the eligibility threshold for incentives on subgoals (interim goals leading up to the ultimate, high goal) improves motivation and adherence more than setting a uniformly highly goal, and a comparison group.

**Trial registration:**

ClinicalTrials.gov NCT05378607. Date of registration: May 18, 2022.

**Supplementary Information:**

The online version contains supplementary material available at 10.1186/s13063-023-07449-z.

## Background

In Sub-Saharan Africa (SSA), 25.6 million people are living with HIV, accounting for 71% of the global total (WHO 2016). Achieving long-term health and reduced likelihood of transmission requires lifelong, high medication adherence, yet low adherence is prevalent among youth, both in the USA and abroad. A recently released U.S. study found only 12% of youth to be virally suppressed [1]. A longitudinal study from South Africa following youth and adults found that adolescents aged 11–19 were approximately 50% less likely than adults to maintain optimally high adherence and 70–75% less likely to be virologically suppressed[2]. In our study among Ugandans aged 15–22, mean ART adherence was 64%, and fewer than 30% were adherent at the clinically desirable rate of 90% or higher [3].

Effective interventions to improve adherence and clinical outcomes for this population are urgently needed, yet a 2018 systematic review found that while there is a sizeable body of evidence on adherence interventions for adults, “current evidence is both sparse and lacking in quality” for adolescents [4]. Another recent review paper [5] found only seven intervention studies targeting youth living with HIV (YLWH), most of which were based in the USA and were small-scale pilot studies with small sample sizes. Clearly, more evidence is needed on what works to improve adherence among youth living in sub-Saharan Africa.

A growing body of work [6, 7] has documented a positive impact of incentives to shift complex health behaviors such as cardiovascular disease prevention [8], sexual behavior [9], and HIV-related behaviors including medication adherence among adults [10], HIV/STI testing [11, 12], and male circumcision [13]. Incentives work by providing a short-term reward for an action that otherwise has only long-term benefits, offsetting “present bias,” or the tendency of many people to act on short-term temptations rather than long-term considerations [14]. They may therefore be particularly appropriate for adolescents who discount the future at higher rates than adults [15]. Recent advances in developmental cognitive neuroscience show that adolescents are more reward-sensitive than adults [16]; adolescents’ socio-emotional brain system associated with impulsivity and risk-taking behavior develops faster than their cognitive control system, with associated problems for self-regulation [17]. Adolescents have consequently been found to derive greater satisfaction from incentives than adults [18].

It remains an open question how best to deliver incentives to improve health behaviors. In existing interventions, incentives typically reward individuals for reaching a certain performance goal, commonly a high threshold set uniformly for all individuals, such as a physical activity milestone [19], adherence of 90% [10], or viral suppression [20]. However, people differ in their levels of baseline performance and capacity to improve their performance. Because traditional, high uniform goals do not reward marginal improvements, they consequently reward those with already healthy behaviors. Individuals who are far from such a high threshold must exert great effort to reach it—and yet any marginal improvements they make will not be rewarded, leading to demotivation. For behaviors which require repeated actions such as (daily) adherence, this structure can discourage effort over time, leading to no or only minimal improvement among those not capable of immediately reaching the high goal. In our own studies, adherence improvements were driven by those with already relatively high initial adherence of about 60–80%. Those with adherence below this level did not benefit from our incentive intervention that set the eligibility threshold at 90% mean adherence, and reported that failing to win the incentives caused demotivation [10]. Adolescents and young adults have the lowest levels of self-esteem of any age group [21] and are therefore particularly susceptible to demotivation. Redesigning incentives to enable those (youth) with low baseline adherence to reach their goals is thus of paramount importance.

New research on behavior change shows that breaking up goals into smaller increments may be the key to reconciling short-term behavior with long-term aspirations [22]. Behavioral economics (BE), and more specifically Prospect Theory, provides a framework to conceptualize goals as reference points [23, 24]. The closer an individual is or gets to a goal, the more motivated they are to achieve it. Conversely, someone far away experiences demotivation [25], resulting in inaction. This concept supports subgoaling, i.e., creating smaller interim goals working towards to the ultimate goal as a solution to overcome the starting problem. Subgoaling takes advantage of individuals working harder when their goal feels more attainable, reinforces motivation by rewarding marginal improvements [26, 27], and boosts task persistence [28]. The approach is most useful when people are doubtful about reaching or are performing far below a distant goal [29, 30].

Allowing participants to set their own interim goals increases agency / ownership [31] and in turn heightens self-efficacy, goal commitment, and performance, compared to assigned (i.e., externally assigned) goals [32–34]. It further avoids the concern that intrinsic motivation may be crowded out by external rewards [35]. This approach also leverages information the participant holds about their capability to reach a goal not evident to the interventionist, i.e., the participant’s private information.

In this study, Goals for Adherence with Low-cost Incentives (GOALS), we will test two approaches to setting interim goals to test the relative contribution of subgoaling and ownership. It is well established in the psychology literature that goals mediate the effect of incentives on performance [36]. Yet evidence is lacking in terms of how to best set the eligibility goals in incentive interventions. GOALS proposes testing externally assigned subgoals (first treatment arm) and participatory subgoals (second treatment arm) with the goal to reach at least 90% adherence. The objective in year 1 is to identify the incentive design most effective for improving adherence. In addition to these interim prize drawings at regular clinic visits, there will be a larger prize drawing at month 12 to make achieving the 90% goal particularly salient. All treatment arms will receive weekly SMS motivational reminders with the goal of keeping incentives and their associated goals at the top of participants’ mind in the weeks between clinic visits. In year 2, the objective is to maintain adherence improvements and achieve viral suppression. Therefore, during the maintenance phase, incentive group participants will be required to remain 90% adherence, with a larger prize drawing for achieving viral suppression at the end of the maintenance phase. Most incentive studies measure behavior change over the short term (less than 6 months) [6]. By explicitly designing incentives to address the dual goals of improvement (year 1) and maintenance (year 2), we provide much-needed evidence on how to sustain adherence over time and prevent performance backsliding. GOALS will measure adherence for 6 months after the intervention ends to assess the impact of the different treatment arms on behavior change once incentives are withdrawn (persistence).

## Methods

### Study design

The study is a parallel group randomized controlled trial, designed as a superiority trial with the primary goal of evaluating whether the GOALS intervention implemented alongside standard care is superior to the standard care alone provided at the Mildmay hospital. Randomization will be performed as block randomization with a 1:1:1:1 allocation. We will recruit participants and monitor their ART adherence using Wisepill devices to measure pill container openings remotely for 3 months. After this period (henceforth “observation period”), we will identify clients exhibiting low adherence, the target population for our intervention. All clients who adhere to less than 90% of their medication in the month before their baseline visit will be recruited for the intervention phase, and randomly assigned to one the GOALS study arms, and those with adherence higher than 90% will be unenrolled.

We will randomize these participants with low adherence into one of four equal-sized study arms for the first year of the intervention (“*Improvement phase*”). In the three treatment arms, participants will be eligible for a prize drawing to win small amounts of mobile airtime of either 500, 5000, or 10,000 Ugandan Shillings (approximately $0.15, $1.50, and $3, respectively) if they reach the adherence goal required in the respective treatment arm. Those randomized to the fourth group will receive usual care as well as weekly text messages, but no incentives. The goals will be set as follows for the different study arms:

1. Assigned subgoals (T1): in this group, the study coordinator will choose the ART adherence target for the participant. This will be done as follows: given an expected four prize drawings during the first year of the intervention, the study coordinator will measure the participant’s baseline adherence and gradually increase the target every 3 months by equal amounts, working towards 90% adherence at the end of year 1. For example, for someone with 50% baseline adherence, the study coordinator would calculate (90% − 50% = 40%; 40%/4 = 10%), and set the adherence targets at the four study visits equal to 60% at month 3, 70% at month 6, 80% at month 9, and 90% at month 12.
2. Participatory subgoals (T2): the participant will choose their own adherence target every 3 months, working towards 90% adherence at the end of year 1. Each target chosen must be higher than the highest adherence ever achieved. For example, someone selected their adherence target in 3 months to be 70%, but they actually achieve 75%. The next target they choose must then be 75% or higher. If a given target was not achieved, the participant can then choose one that is equal or higher to the one chosen previously.
3. Fixed goal (T3): the study participant must reach a fixed (i.e., non-varying) target adherence level of 90% at each study visit; this arm corresponds to how adherence interventions typically are implemented.
4. Control group (T4): will receive the usual standard of care offered at Mildmay Uganda.

The three treatment groups will receive weekly motivational messages and a reminder of their upcoming prize drawing. Weekly SMS messages will help maintain contact with study participants and enable them to become accustomed to receiving messages from the study for potential remote prize drawings (given the evolving COVID situation and corresponding clinic changes towards visits that are more spaced out, we expect some prize drawings to be done in person, and others remotely as further detailed below). In contrast, the control group will only receive the motivational portion of the text messages (without the reminders of the possibility of winning prizes) as an attention control due to the potential beneficial effects of text messages.

After the first year of the study, we will evaluate participants in the treatment groups to determine those who achieved high adherence of at least 90% (high adherers) and those who did not (low adherers).

**Fig. 1:**
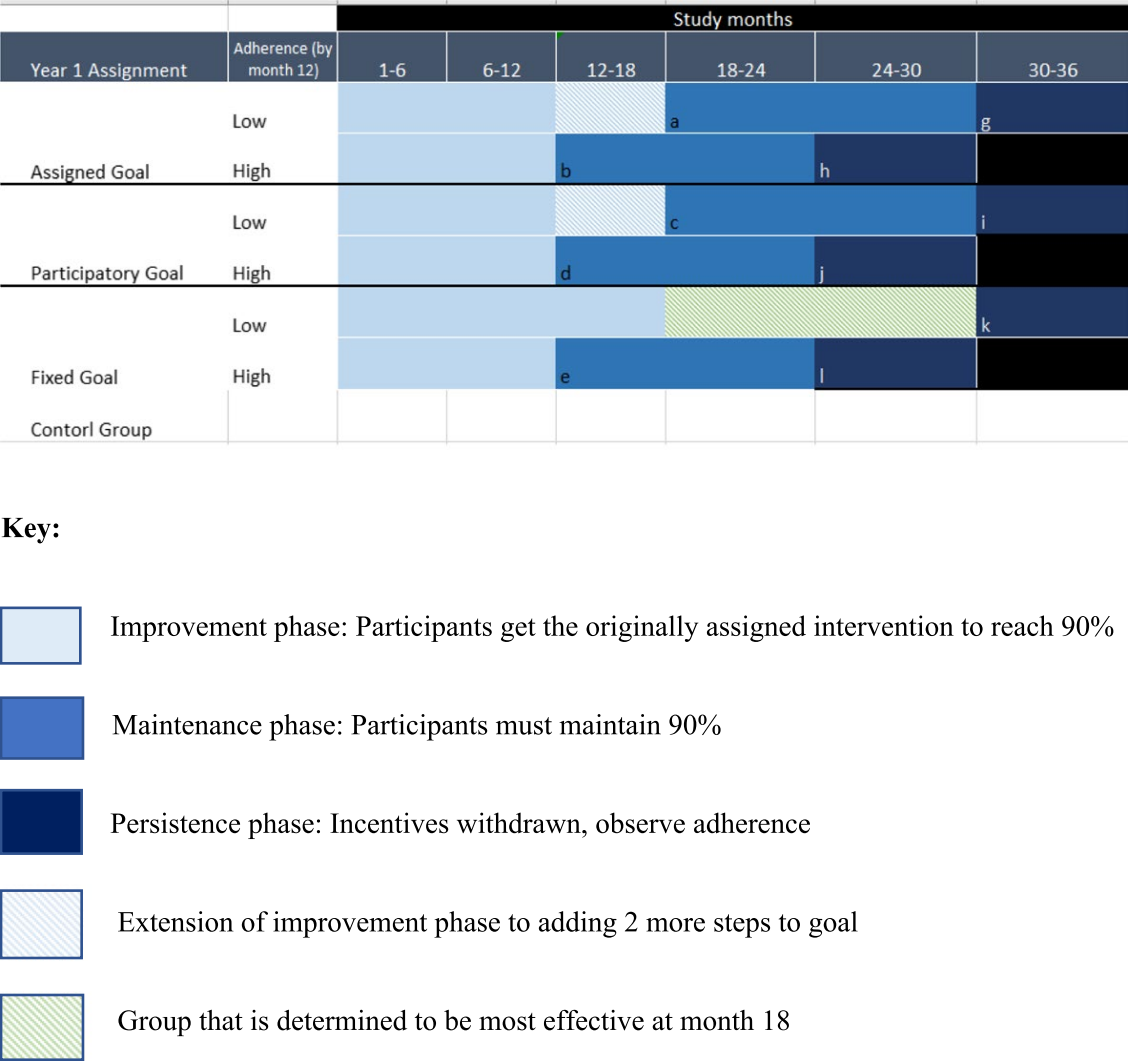
Table showing different phases of the participants in different groups throughout the study

Figure 1 below shows that in year 2, high adherers will progress to the “Maintenance phase” between months 12 and 24 where we will assess the intervention’s effectiveness in maintaining at least 90% adherence. They will have the chance to win prizes every 3 months if they maintain their adherence at or above 90% throughout this phase. They will then move to the “Persistence phase” between months 25 and 36 to assess the treatment arms’ impact on long-term adherence once incentives are removed.

Low adherers will be given an additional 6 months in the “Improvement phase” between months 12 and 18. During this time, low adherers in the participatory subgoals group will have one more opportunity to set their personal target; they will be encouraged to set a target at month 15 such that they would achieve 90% by month 18. Low adherers in the assigned subgoals group will receive two more pre-chosen targets starting from their adherence at month 12 in equal increments up to 90% (e.g., if month 12 adherence is 80, their target is 85 for month 15 and 90 for month 18). The maintenance phase for the low adherers in the assigned goal and participatory goal groups will begin at month 18 for 12 months.

Low adherers in the fixed goal group will maintain their target of 90%. At month 18, we will identify the treatment arm with the highest effectiveness and move low adherence participants in the fixed goal arm to that arm to give them an opportunity to also benefit from the intervention. These participants will continue in the improvement phase between months 18 and 30, while assigned subgoals and participatory subgoals participants move to the maintenance phase. Low adherers across in the participatory and assigned goals group will complete their “Persistence phase” of the study in months 30–36.

All participants in the treatment groups will be eligible for a larger prize drawing at the end of their maintenance phase if they achieve viral suppression, defined as a viral load of 200 copies/mL or less.

Follow-up surveys will be conducted every 6 months for 24 months, and a cost-effectiveness analysis will also be performed after 36 months to help determine the intervention design’s potential for sustainability and scale-up.

### Study sites

The study will be conducted at Mildmay Uganda, an NGO with headquarters in Uganda’s capital, Kampala. Mildmay Uganda specializes in the provision of comprehensive HIV and AIDS prevention, care, and treatment services. Mildmay Uganda provides quality outpatient and inpatient HIV care and trains health care workers throughout Uganda and the region in the provision of such care. Mildmay serves over 105,000 patients (15,000 at the main site in Lweza and over 95,000 at supported health facilities in 8 districts in Central Region of Uganda). The main-site facility has a well-trained, experienced team of clinicians and health workers, and modern laboratory infrastructure with ability to do virology and other tests. The Mildmay Uganda laboratory is accredited by the South African National Accreditation System (SANAS) for International Organization for Standardization (ISO) 15,189:2012 for medical laboratories and also acts as a national back up laboratory for the government of Uganda Central Public Health Laboratories. Some of the services provided include the following: HIV counseling and testing; pediatric and adult HIV prevention, treatment and care services; sexual and reproductive health services; diagnostic (laboratory) services and radiology; rehabilitative services (nutrition, physiotherapy, occupational therapy); safe male circumcision (SMC); ophthalmic; and dental care. Of the 15,000 patients served at the main site in Lweza, 11% are children below 18 years, 65% are female, and 100% of all clients in care are on ART. Mildmay is one of a growing number of facilities with a well-established electronic medical records system in Uganda.

### Sampling and participants

We expect to enroll 1256 individuals for the observation phase of the study that serves to measure baseline adherence. Monitoring baseline adherence will ensure that individuals being randomized to the interventions are indeed those in need of adherence support, i.e., have relatively low baseline mean adherence of less than 90%.

We arrived at this starting sample size in the following way. For the primary outcome of adherence, a sample size of 140 participants in each arm (*n* = 560 total) will be able to detect an 8 percentage point (pp) difference in mean adherence between each treatment arm and control, and compared to each other. The corresponding difference at month 24 is 9 pp. This assumes mean control group adherence of 62%. In order to reach this required sample size at year 3, we assume 6% attrition per year over 2 years of the study (1.06^2 = 1.1236) which results in a sample size for the beginning of GOALS recruitment to be 629 (560 × 1.1236). To ensure an even number across the four study arms, we reduce this target from 629 to 628. We then assume that 50% of those who complete the adherence observation phase are eligible for the GOALS intervention. We therefore need a starting sample size of 1256 ((628/50) × 100). All assumptions surrounding attrition rates and rates of adherence are based on data from PI Linnemayr’s previous study at the same site.

#### Inclusion criteria

For a participant to be eligible for the 3-month observation period preceding the GOALS intervention, they must meet the following requirements:

- Aged 15–30 inclusive
- Receiving ART medication from Mildmay for more than 3 months
- Has regular access to a mobile phone (less than 5 out of 7 days)

In order for participants to be eligible for the GOALS intervention after the observation period, the participant must meet the following additional requirement:

- Have a mean adherence below 90% in the last month of the observation period

#### Exclusion criteria

Exclusion criteria for the 3-month observation period preceding the GOALS intervention and for the GOALS intervention:

- Is not willing or able to use the Wisepill device when taking ART medication
- Has mental health problems that require immediate treatment (e.g., psychotic symptoms), a diagnosed mental disorder that would limit the ability to participate (e.g., dementia), or cognitive impairments that result in a limited ability to provide informed consent.
- No longer has access to a phone (less than 5 out of 7 days), if phone access changed over the course of the 3-month observation period
- Participating in another study
- Unable to read

### Randomization

Random treatment assignment will occur after participants are recruited, and before they complete a baseline survey using a 1:1:1:1 ratio. Randomization will be done using sequential assignment while stratifying by baseline adherence measured during the observation period (0–54%, 55–71%, 72–82%, 83–89%). Stratification will be performed to increase power and achieve better balance than in a simple random draw. A randomization schedule will be established ex ante and sequential participants within a stratum randomized accordingly. The randomization schedule will contain blocks of four possible randomizations to the three treatment arms or the control group, with the order of the four possibilities randomized each time a new block begins. We will use the *ralloc* function in Stata 17 to generate the randomization lists, which will be uploaded to SurveyCTO and programmed to randomize participants on the spot.

The observation phase lasts approximately 3 months, after which adherence-based eligibility is determined, and all recruited participants will complete a baseline survey. They will be informed of their assignment to either one of the three intervention arms or the control group *after* completing the baseline survey. Participants who provide informed consent will be asked to participate in the baseline survey *before* the treatment assignment is revealed to make sure group assignment does not influence the answers given.

Participants cannot be blinded to their treatment status and neither can interviewers. The data analyst who will conduct the impact analysis will be blinded to treatment assignment.

As the only blinded party is the analyst, and the blinding occurs to avoid any bias of the analyst to find a statistically significant effect for the intervention group(s), we cannot think of a justified reason for unblinding the analyst.

### Design

**Fig. 2:**
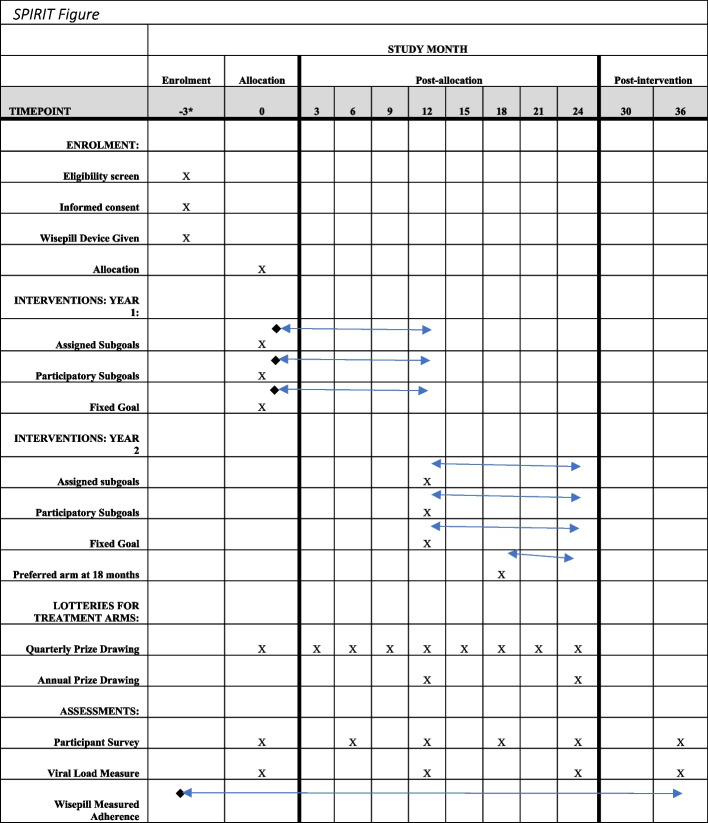
SPIRIT figure

The study will use three intervention arms and a control arm. The intervention arms will all provide mobile airtime as incentives, but the incentives will be based on different eligibility criteria in the different arms. We will collect ART adherence data continuously for 3 months prior to the intervention, for 24 months after the intervention begins, and for 12 months after the intervention ends for all participants using Wisepill devices. We will acquire routine viral load measures for all participants throughout the study, which will be performed roughly every 12 months as per clinic and Uganda Ministry of Health guidelines. We will also conduct a baseline survey, and then surveys every 6 months for 24 months for all participants. See Fig. 2: SPIRIT figure for a detailed description of the timing of study activities.

This study protocol uses the SPIRIT reporting guidelines [37].

### Procedures

#### Intervention components

All participants in the treatment arms will be eligible for a quarterly prize drawing if they meet the goal that corresponds to their treatment assignment, and for an annual prize drawing if they show 90% adherence by the time they reach month 12 (as measured by reaching 90% in the last 3 months of month 12).

#### Quarterly prize drawings

Participants in the three treatment arms will be eligible to enter an in-person or remote prize drawing for a mobile airtime reward every 3 months if they meet their target adherence. In-person prize drawings will be done if the date of the drawing coincides with the participants’ regularly scheduled clinic visits; otherwise, they will be done remotely. Wisepill records adherence remotely, so the study team can monitor adherence without an in-person visit. Remote prize drawings will be conducted automatically by the computer, and the results and airtime reward texted to the participant over the phone.

### Assigned subgoal eligibility

Participants in the assigned subgoals arm will be eligible for the quarterly prize drawing if they reach their assigned subgoal in the respective quarter. The goal will be assigned based on observed baseline adherence, which will be deducted from 90%, and divided by four to get at 4 equal, relatively small, increasing steps. For example, someone with 50% baseline adherence would be asked to reach 60% at month 3, 70% at month 6, 80% at month 9, and 90% at the end of the first intervention year.

### Participatory subgoal eligibility

Participants in the participatory subgoals arm will be eligible for the quarterly prize drawing if they reach the goal they choose for themselves in the respective quarter. The only restrictions are that each goal has to be equal to or higher than the previous one, and that at month 12 90% adherence is reached.

### Fixed goal eligibility

Participants in the fixed goal arm will be eligible for the quarterly prize drawing if they reach 90% adherence in the respective quarter.

Patients scheduled for the in-person prize drawing will also be required to complete a prize drawing survey after they participate in a prize drawing. This survey will measure how feedback given on the achieved versus planned ART adherence influences motivation for future performance and perceived attainability of the next goal. Target, distance, and valence influence attitudes and motivation towards future goals.

#### Annual prize drawings

Participants in all three treatment arms will be eligible to enter a larger prize drawing at the end of year 1 if they reach 90% adherence over the course of 12 months.

#### Wisepill procedures

All participants will be given a Wisepill device upon recruitment into the observation period of the study. At the time they receive the device, they will be instructed in how to refill the pill box. The team will only monitor adherence to one antiretroviral as studies show that rates of adherence do not differ significantly across a patient’s medications.

Participants will be asked to use the Wisepill device continuously throughout the study and to notify the study coordinator if their provider changes their regimen during the study (which may be a real possibility, in particular for the youths on prophylaxis). They will be asked to remove only one dose at the time of ingestion and will also be informed that the data recorded will not be shared with clinicians. The team will carefully discuss the restrictions associated with the device with the patient and solutions to potential concerns that are acceptable to both the patient and the project will be generated. We will review the instructions and restrictions with the participant at each successive clinic visit.

The 3-month period will also allow individuals to incorporate the device into their daily life and to test and troubleshoot its use prior to beginning the intervention. During this time, we will monitor their adherence. Participants who are unable to use the device consistently will be excluded from the study after careful discussion of the reason for the low observed adherence with the patient (to avoid excluding participants with truly low adherence that is not related to an inability or unwillingness to use the Wisepill device). We will also exclude participants who have adherence above 90% during this period to target the intervention at those with low Wisepill-measured adherence (as those over 90% adherence have little room and/or need for improvement).

### Intervention modifications

Participants will be discontinued from the study if they experience serious adverse events related to the intervention. Participants will also be discontinued from the study at their own request, if they are not in a position to adhere to study protocols which include the use of Wisepill devices to monitor their medication intake.

### Strategies to improve adherence to intervention protocols

Intervention implementation will be closely monitored via inspecting routine data collected through surveyCTO, conducting regular data quality checks, and providing the study team up to date data to facilitate implementation. Progress trackers are based on incoming data, which tracks the number of people who have begun each phase of the intervention, those randomized to each group, and those who completed each study activity. Study coordinators are provided a study activity tracker, which updates weekly, to inform them of upcoming in-person prize drawings that coincide with clinic visits. Remote prize drawings are conducted via the Telerivet SMS platform. For both prize drawing messages and weekly SMS messages, the study team tracks message sending success rate on a weekly basis and resends messages that were not successful the first time. Weekly team calls are also conducted across study coordinators and PIs to debrief progress and challenges. Site investigator MO monitors implementation fidelity regularly; in addition, PIs Huang and Linnemayr visit several times a year to observe study activities and troubleshoot issues.

### Study timeline

The SPIRIT figure above shows the study timeline.

#### Recruitment

During recruitment for the observation phase, the client will be given a Wisepill device. During this recruitment visit, the study coordinator will consent the participant and explain the reason for and conditions of using the Wisepill device. Participants will be compensated 10,000 USh for their time.

As dates of the next appointment are readily available from the electronic medical records system, the clinic system will allow us to print out weekly lists of eligible clients together with the date they are expected at the clinic, and the date they are scheduled for their next viral load test.

We will consent and enroll 4–6 clients per day (some clients may refuse participation, though this has been rare in our previous studies) during the 9- to 12-month recruitment period. Based on the large clinic population and our previous experience, we expect to easily recruit the 1256 clients within a 12-month period but allow for sufficient extra time to accommodate any delays due to public holidays and other reasons for clinic closures or other delays.

### Baseline survey and randomization

When the client returns for their next scheduled clinic visit after the observation period (after about 3 months) and is eligible based on their observed adherence, we will conduct the baseline survey, reveal treatment assignment to either the control or one of the three intervention groups, and begin the intervention. In scheduling these return visits, we will aim to schedule them to coincide with a scheduled clinic visit so that participants do not have to come to the clinic for the sole purpose of study activities. However, participants with next scheduled visits longer than 3 months will be messaged or called to come back at the 3-month mark.

The baseline survey contains the following information:

- Demographics and socioeconomic status, including age, gender, education, relationship status, employment type and status, income, housing, economic shocks, and household composition;
- HIV status and clinic visits, e.g., respondents will be asked whether they have disclosed their status to members they live with and the number of those who have HIV/AIDS, their primary caregiver, the person that ensures they take their ART medication, and their clinic visit habits and expenses;
- Adherence self-efficacy, i.e., reasons for non-adherence or failure to seek care (we will use an 11-item modification developed by the ACTG, that asks participants to indicate whether listed items were reasons for their not taking medication in the previous month or seeking care, such as “when the drugs make you feel bad,” or “when your daily routine is interrupted,” or “lacked resources).
- Participants’ subjective experiences related to taking medications [38] using the Intrinsic Motivation Inventory. The survey will also collect information related to behavioral economics biases such as present bias or risk preferences.

### Follow-up surveys

Follow-up surveys will be conducted at month 6, 12, 18, and 24 post baseline. These assessments will allow us to collect several data points for each participant on mediators or moderators that we believe may be influenced by the intervention (e.g., cognitive and motivational factors). Participants will be compensated 30,000 UGX for their time every time they complete a survey questionnaire.

### Retention

To promote participant retention and complete follow-up, the study has aligned its activities with the participants’ clinic visit dates. By doing this, the study aims to ensure that participants are not only coming to the clinic for study-related activities but also for their routine clinical visits. This approach is expected to reduce the burden on participants, minimize missed appointments, and increase compliance with the study protocol. Participants who discontinue the study will return their Wisepill devices and we will not be collecting outcomes data.

### Outcomes

#### Primary outcome

Outcome 1: Mean adherence to ART (Time Frame: adherence over a 12-month period continuously tracked remotely): Wisepill devices monitor the date and time of all device openings to retrieve ART medication, allowing ART adherence to be tracked continuously on the Wisepill server. Mean adherence will be coded as the number of ART doses taken / number of doses prescribed, with adherence being capped at 100% each day to avoid multiple openings on a given day artificially inflating mean adherence.

Outcome 2: The fraction of clients with adherence of 90% or more (Time Frame: continuously tracked remotely).

Outcome 3: Probability of reaching adherence target (Time Frame: tracked every 3 months corresponding to routine prize drawing opportunities). Probability that participants in the treatment groups reach their assigned target (T1), self-selected target (T2), or high fixed target of 90% (T3).

#### Secondary outcomes

Outcome 1: Fraction of clients with treatment interruptions of more than 48 h (Time Frame: Continuously tracked remotely).

Outcome 2: Suppressed viral load (viral load ≤ 200 copies/ML) among participants (Time Frame: At baseline and month 24): Viral load will be chart abstracted from clinic data. They are typically done once a year as part of routine clinical care.

Outcome 3: Retention in Care, chart abstracted from clinic data. First, we will use the binary definition of retention from the Health Resources and Services Administration HIV/AIDS Bureau as at least 2 clinic visits separated by 90 days in the previous 12 months. Second, we will use the more detailed measure outlined by Lee et al. [12] to create a variable that categorizes clients as having different levels of retention in care that provides more granular information about retention. We will define a client as fully retained in care if they attended all scheduled appointments over the previous 12 months (usually 4 or 5 total appointments). We will then create three different levels of care disengagement: missed one appointment but not more than 6 months without a visit, missed 2 appointments or 6–9 months without a visit, and missed 3 or more appointments or 9–12 months without a visit.

### Sample size and power

We have calculated the size of effects that our sample will be able to detect with 80% power (2-tailed test) with regard to outcomes at months 12 and 24, and assuming 10% attrition every year (we observed 8% attrition for our pilot study, so this is a conservative estimate). For the *primary outcome of adherence*, a sample size of 140 participants in each arm (*n* = 560 total) will be able to detect an 8-percentage point (pp) difference in mean adherence between each treatment arm and control, and compared to each other. The corresponding difference at month 24 is 9 pp. This assumes mean control group adherence of 62%, based on our pilot data. For the *secondary outcome of viral suppression*, we use a conservative estimate of about 70% of clients in the control group showing suppression based on discussions with the Mildmay team. Our sample size of 140 participants in each of the three arms will be able to detect about a 10 pp difference at month 12, and 11 pp at month 24. These represent conservative estimates of minimum detectable effect size that do not account for stratified randomization that we will perform to increase power, and to achieve better balance compared to a simple random draw [39].

### Data analysis

Our primary analyses will be intention-to-treat analyses, with secondary analyses involving study completers only.

#### Estimating impact on primary outcomes

To estimate the impact of our interventions on primary outcomes, we will use an ordinary least squares Linear Probability Model. In the context of experimental evaluations, linear probability models provide unbiased estimates of program impact and correctly estimate standard errors (Gomila, 2020; Deke, 2014) [40, 41]. We will estimate an unadjusted model and a model that includes the following prespecified covariates to adjust for baseline characteristics and improve precision: age, education, sex, strata fixed effects, baseline WHO disease stage, baseline viral suppression, duration on ART, socioeconomic status as measured by the Poverty Probability Index for Uganda, and HIV disclosure status.

Mean adherence to ART and the fraction of clients with adherence of 90% or more will be analyzed with the following adjusted and unadjusted models:

Unadjusted:

1 $${y}_{it}={\beta }_{0}+{\beta }_{1}assigne{d}_{it}+{\beta }_{2}parti{c}_{it}+{\beta }_{3}fixe{d}_{it}+\overrightarrow{{\beta }_{4}}{\overrightarrow{S}}_{i}+{\epsilon }_{i}$$

Adjusted:

2 $${y}_{it}={\beta }_{0}+{\beta }_{1}assigne{d}_{it}+{\beta }_{2}parti{c}_{it}+{\beta }_{3}fixe{d}_{it}+\overrightarrow{{\beta }_{4}}{\overrightarrow{S}}_{i}+{X}_{it}\alpha +{\epsilon }_{i}$$

${y}_{it}$ will be analyzed in two ways: as a continuous variable denoting mean adherence as the share of the prescribed bottle openings that were opened by individual $i$ on week $t$, and as a binary variable = 1 if the mean adherence exceeds 90% and = 0 if it is below 90%. In this model, the ${\beta }_{1}$ represents the impact of assigned subgoals on the share of pills taken in a given week, ${\beta }_{2}$ represents the impact of participatory subgoals on the share of pills taken in a given week and ${\beta }_{3}$ represents the impact of fixed goals on the share of pills taken in a given week. ${\overrightarrow{S}}_{i}$ represents strata fixed effects. Equation 3 includes the term ${X}_{it}$, which represents the same covariates that are predictive of adherence.

The “Maintenance” and “Persistence” phases of the study will be analyzed for the number of months the participant spends in each phase. For the improvement phase, we will focus only on the month preceding month 12, as for that phase each participant in the participatory and assigned subgoals groups will display a different subgoaling path, and the only goal they share is to reach 90% adherence by month 12 (and eligibility for the prize drawing is measured for the 30 days preceding the month 12 visit).

### Subgroup analysis

We will also do some exploratory subgroup analyses based on the following characteristics:

- High vs. low baseline adherence
- High vs. low baseline intrinsic motivation to take treatment
- High vs. low baseline self-efficacy
- High vs. low frequency in usage of sending and receiving messages through their mobile phone

### Standard errors

We will estimate Huber-White robust standard errors in all analyses. All standard errors will be clustered at the individual level.

### Adjusting for multiple hypotheses

*P*-values will be adjusted for multiple hypothesis testing for all secondary outcomes by applying a false discovery rate adjustment (FDR) following Benjamini et al. (2006) [42]. In particular, we will use the sharpened *q*-values discussed in Anderson (2008) [43]. This correction will control for the expected proportion of type 1 errors.

### Data management

Existing clinic identifiers will be used as unique study identification numbers during data collection. Consent forms will bear the name and signatures of study participants, but all other information (such as viral load tests and Wisepill measurements) will be collected using these unique clinic identifiers. The study team in Uganda (one team leader, two lead interviews, and three supporting team members) will be in charge of collecting all of the data and carrying out the in-person intervention. All survey data will be collected with computer-assisted software (SurveyCTO) using a tablet. The survey data will be uploaded to the password-protected study computer at the end of each day (at which point they are then automatically deleted from the device) and saved in an encrypted folder on that computer.

All datasets and files with identifiers will always remain encrypted through a zipped folder or by using BoxCryptor within DropBox, an online data portal that can only be accessed using a secure password. Adherence to HIV antiretrovirals will be monitored using a Wisepill device. Data from these devices will be uploaded remotely to an encrypted server using a sim card embedded in the Wisepill device in real time. Participant adherence data will then be downloaded and stored securely via BoxCryptor and will only be identified via an alpha-numeric clinic ID. SMS messaging data will be downloaded from the messaging platform, Telerivet. Text messaging data will not include sensitive or private information, such as the participant’s name or current HIV status at any point. Downloaded datasets will include text messages sent and received, time, and date of messages. For any data exports containing participant’s phone number, we will ensure this always remains in an encrypted folder using BoxCryptor within DropBox.

Paper copies of consent forms will be stored and locked at the Mildmay RAND office in Kampala, and access will only be granted to key personnel and the study PIs. Any published material will not contain participants’ identifying information. There is no formal data monitoring committee since the trial was deemed minimal risk, but data monitoring will occur through weekly checks by the study team in the USA, and an independent study monitor.

### Handling missing data and attrition

Missing data have been a minor issue in our previous studies with the same study population and outcomes. Attrition has been well under 10% per year. However, when subjects drop out, we will fit multiple logistic regression models to assess whether this dropout is random. If it is not, we will construct “nonresponse” weights using logistic regression that correct for dropout by assigning weights to continuing subjects that are inversely proportional to the predicted probability of the subjects’ continuing to the time period in question. Analyses will incorporate design effects from this weighting in the calculation of standard errors and tests of significance. In addition, we will present sensitivity tests regarding changes in outcomes when excluding those with missing observations to give a fully transparent picture of the data.

### Oversight and monitoring

#### Data monitoring formal committee

The study is a clinical trial with a low risk of harm. Therefore, we appointed a single independent clinical psychologist who is an expert in treating depression in people with HIV as the monitor instead of using a Data Monitoring Committee (DMC). The monitor will receive reports every 6 months on subject enrollment, retention, adverse events, and reasons for dropouts and will review the reports and make recommendations regarding the study’s continuation, modification, or termination. All the communication from the independent monitor will be shared with the institutional review boards and NIH. The monitor is independent of the sponsor and has no competing interests.

### Interim analysis

As described in the “Study design” section, interim analysis will be conducted at month 12 to classify participants into those who have reached 90% and those who have not. At 18 months, we will analyze impact results across study participants to determine the treatment arm with the highest effectiveness; participants in the fixed arm who have not reached 90% adherence will then be moved to this arm.

### Adverse event reporting and harms

While we are doing everything to avoid that a study participant is at increased risk because of any study-related activity, we will be very careful in tracking any potential negative events experienced by all study participants. Adverse events relating to ancillary and post-trial care may encompass both physical and psychological harms. The study coordinator will be experienced and trained to recognize risks or crises that require referrals. Team members have established procedures and guidelines to respond to risk disclosures and crisis situations among participants. If there are indications during the study visits that a participant poses a risk of suicide or to harm him/herself, the interviewer will stop the session and explain to the participant that they would like an on-site Mildmay mental health counselor to speak with the participant about the situation. The counselor would then assess the risk for potential harm and the appropriate action in terms of evaluating the client’s need for mental health services and notifying appropriate authorities. This assessment would be done as soon as possible and before the client leaves the premises to the extent possible. A serious adverse event report will be filed, if necessary. This will all be done immediately if possible and certainly within 24 h.

Anything that looks like it could be an adverse event will be brought to the attention of the local and study PI as each case needs to be investigated. Any unexpected, serious adverse events that occur during the course of this investigation and follow-up period will be reported by telephone by one of the PIs, Dr. Linnemayr or Dr. Huang, within the next business day to the study IRBs and the independent study monitor. The telephone report will be followed within 3 business days by a written report, which will contain the following: subject’s ID#, the title and date of serious adverse event, and narrative explanation (e.g., how the research staff was notified of the event, dates of consent, study screening for inclusion/exclusion, whether the participant participated in the intervention or was in the control condition, dates and circumstances of the hospitalization/death, and participant status at last clinical or research contact). In consultation with the IRBs, the PI will address whether there is a need to redesign or amend the protocol, and/or to inform current and future subjects of a change in description of risk (e.g., in consent form and protocol).

Except for adverse events, we do not expect any reasons for discontinuing the intervention. As per our IRB protocol, participants are free to discontinue the study at their own discretion. Due to the low-risk nature of the intervention, there will be no interim analyses, or stopping rules.

### Auditing

The Mildmay Uganda Research Ethics Committee and the Uganda National Council for Science and Technology will conduct an independent audit of the study once every year. The procedures for audit will include a site visit, document reviews, participant record reviews, and interviews with study coordinators. The auditors will also review compliance with regulatory requirements and ethical principles, as well as the accuracy and completeness of data collection and reporting.

### Dissemination of results

Study findings will be disseminated to researchers and clinicians via peer-reviewed publications and sharing of findings at conference presentations. Authorship of published papers will follow established guidelines for defining the level of contributions that warrant authorship. These findings will also be relevant to local Ugandan and global communities with an interest in understanding underlying behavioral mechanisms that affect HIV treatment adherence. We will share these findings with senior level officials at the Ministry of Health and at Mildmay Uganda, so they can formulate appropriate policy in line with the national recommendations on annual viral load screenings for HIV-positive patients.

The full protocol will be made publicly available. Participant-level dataset and statistical code will also be made available upon publication of the impact results.

## Discussion

### Potential impact and significance of the study

Treatment adherence is critical to the success of ART and is largely determined by behavior. Studies using incentives have shown great promise to improve ART adherence, but there is little evidence on how to best structure these incentives, and in particular how to set the eligibility criteria to qualify for rewards, with the typical approach being to set a relatively high, same adherence goal for all participants. In this study, we test three different ways of structuring incentives for short-term improvement, as well as medium-term maintenance and persistence that allow for more incremental steps to reach 90% adherence after 1 year to avoid potential demotivation for those with low adherence (and hence a large step to reach 90% adherence) that we observed in previous studies.

We target these novel behavioral interventions to improve ART adherence to treatment-mature clients, i.e., the increasing number of people who have been on ART for a number of years who battle flagging motivation to consistently adhere to their medication. This study will be one of the first we are aware of that tests an intervention designed to increase the motivation of treatment-mature clients through the chance to participate in prize drawings based on observed adherence. The findings of this study will provide unique insight into the underlying behavioral mechanisms that affect HIV treatment adherence, which can be exploited to improve adherence in a variety of settings.

If found successful, the interventions in this study are readily scalable, while the Wisepill devices used to measure adherence are relatively expensive, they can be re-used for multiple patients, and their price can be expected to come down over time. Furthermore, there are alternative ways of ascertaining adherence (such as through asking the participant to send a picture of their medication at the time of swallowing) that can be implemented at very low cost. Irrespective of the mode of implementation, our study tests out different conceptualizations of implementing incentives, with immediate relevance for a range of other conditions (i.e., most conditions requiring consistent medication adherence) and other health behaviors (such as weight loss, where relatively frequent, incremental steps may be more motivating than a relatively large weight loss required at a point far in the future).

### Limitations

This study has several limitations. First, the study will only include 640 patients in one clinic in Uganda. Although our sample size is well powered to detect clinically important effects, it is not clear that our results will extrapolate to other areas of Uganda or other countries. Second, although adherence is measured using Wisepill devices, which is currently one of the most accurate ways to measure adherence, we may not be able to exclude the possibility that some participants consciously manipulate the pill bottle openings to increase their chances of receiving the incentives. Third, while we conceptually test out different ways of structuring incentives, there is little guidance on the “right” size of incremental steps to effectively lead participants to clinically meaningful adherence levels, and further research is required to determine their most effective magnitude.

### Trial status

The trial registration number is NCT05378607. Registered May 18 2022, https://clinicaltrials.gov/ct2/show/NCT05378607. The study start date was April 25, 2022. The protocol reported here is dated May 5, 2023. Patient recruitment is currently under way. The primary completion date is 8/ 31/ 2026 and study completion date is 11/31/2026.

### Supplementary Information

**Additional file 1. **Spirit checklist

**Additional file 2. **Appendix

## Data Availability

External researchers interested in investigator data, survey instruments, and other research methodology and procedures will be able to obtain this information through collaborative agreements (e.g., data use agreements) with the Principal Investigators and Co-Investigators, as required by NIH’s data sharing policy.

## Abbreviations

ART: Antiretroviral treatment
HIV: Human immunodeficiency virus
STD: Sexually transmitted disease
BE: Behavioral economics
BEST: Behavioral Economics Incentives to Support HIV Treatment Adherence
NGO: Non-governmental organization
SMC: Safe male circumcision
CD4: Cluster of differentiation 4
ACTG: AIDS Clinical Trials Group
ICER: Incremental cost-effectiveness ratio
SSA: Sub-Saharan Africa
YLWH: Youth living with HIV
SANAS: South African National Accreditation System
ISO: International Organization for Standardization
