# Supplementary material for: Goals for Adherence with Low-cost Incentives (GOALS): a protocol for a randomized controlled trial evaluating the impact of small airtime incentives on ART adherence among young people living with HIV in Kampala, Uganda
Source: Trials. 2023 Aug 9;24:511. doi: 10.1186/s13063-023-07449-z (PMC10410910; doi:10.1186/s13063-023-07449-z)
Supplement: Supplementary file 2 — Additional file 2. Appendix [file 13063_2023_7449_MOESM2_ESM.docx]

# Appendix

## Appendix 1: Administrative information

| Title | Goals for Adherence with Low-cost Incentives (GOALS): A protocol for a randomized controlled trial in Kampala, Uganda |
| --- | --- |
| Trial registration | **Name of registration:** ClinicalTrials.gov  **Trial registration number:** NCT05378607  **Date of registration:** May 18, 2022  **URL of trial registry record:** https://clinicaltrials.gov/ct2/show/NCT05378607 |
| Protocol version | **Issue date:** 5 May 2023  **Version number:** 01 |
| Funding | The study is funded by the National Institutes of Mental Health (R01) 5R01HD104555-02 (mPIs Huang and Linnemayr). |
| Author details | Sebastian Linnemayr, RAND Corporation, email: [slinnema@rand.org](about:blank)  Haijing Crystal Huang, IDinsight, email: [crystal.huang@idinsight.org](about:blank)  Zachary Wagner, RAND Corporation, email: [zwagner@rand.org](about:blank)  Faith Kemunto Onkundi, IDinsight, email: faith.kemunto-onkundi@idinsight.org  Barbara Mukasa, Mildmay Uganda, email: [barbara.mukasa@mildmay.or.ug](about:blank)  Mary Odiit, Mildmay Uganda, email: mary.odiit@mildmay.or.ug  **Corresponding Author:** Crystal Huang, IDinsight, email: [crystal.huang@idinsight.org](mailto:crystal.huang@idinsight.org)  **Authors' contributions**  SL and HCH designed the study and led all aspects of the protocol development. ZW drafted the initial protocol manuscript. FKO edited and refined the protocol manuscript. BM and MO advised on the study design and intervention implementation. All authors read and approved the final manuscript. |
| Name and contact information for the trial sponsor | **Trial sponsor:** [Eunice Kennedy Shriver National Institute of Child Health and Human Development (NICHD)](https://www.nichd.nih.gov/)  **Address:** Office of Communications, 31 Center Drive, Building 31, Room 2A32, MSC 2425, Bethesda, MD 20892-2425 **Phone:** 1-800-370-2943 **E-mail:** [NICHDInformationResourceCenter@mail.nih.gov](mailto:NICHDInformationResourceCenter@mail.nih.gov) |
| Role of sponsor | The funder and sponsor played no role in the study design; collection, management, analysis, and interpretation of data; writing of the report; and the decision to submit the report for publication. |
| Responsibilities of the committee | *Stakeholder and Public Involvement Group (SPIG)*  Not Applicable: There is no SPIG for this study.  *Principal investigators*   - Overall design and management of the study - Study planning - Reviewing progress of the study and agreeing changes to the protocol - Responsible for trial Masterfile - Budget administration and contractual issues with the clinic - Publication of study report   *Study associate*   - Maintenance of data entry system - Data verification - Managing study coordinators   *Study coordinators*   - Recruitment and randomization of patients - Conducting in-person interventions |

## Appendix 2: Informed Consent forms

### **2.1 Study 1 Guardian Consent form**

Study ID ……………… Clinic ID …………..

**Consent Form for Parent or Guardian Study 1**

**“Goals for Adherence with Low-cost Incentives (GOALS)”**

**Lead Principal Investigator:**

Dr. Sebastian Linnemayr, PhD

Institution of affiliation: RAND Corporation

Address: 1776 Main Street, Santa Monica, CA 90407-2138, US

Phone contact: +1 310 393 0411 ext 6734

Email: slinnema@rand.org

**Principal Investigator:**

Dr. Crystal Haijing Huang, PhD

Institution of affiliation: IDinsight

Address: 44 Tehama Street, San Francisco, CA 94105, US

Phone contact: +1 202 436 1411

Email: crystal.huang@idinsight.org

**Site Principal investigator:**

Dr. Barbara Mukasa

Institution of affiliation: Mildmay Uganda

Address: P.O. Box 24985, Kampala; Uganda

Phone contact: +256 312 210 200 or +256 772 700 816

Email: barbara.mukasa@mildmay.or.ug

**Sponsor:** National Institute of Child Health and Human Development (NICHD), USA

**Collaborating Institutions:** IDinsight, The RAND Corporation, Mildmay Uganda

**INTRODUCTION**

Hello, my name is [insert name of the Study Coordinator]. I work at Mildmay Uganda that in collaboration with the RAND Corporation, and IDinsight, is conducting a study at Mildmay Uganda. The purpose of this study is to understand antiretroviral therapy (ART) medication adherence among young clients at Mildmay Hospital. This study has been approved by an accredited Ugandan-based Research Ethics Committee, the Mildmay Uganda Research Ethics Committee.

Before you decide whether or not your child will participate in this study, we would like to explain what the study is about, how it may help your child, any risks to your child, and what is expected of your child. After I talk about the details of the study with you, I will ask you if you want your child to be part of the study and if yes, you will be asked to sign an Informed Consent form on their behalf. This process will take approximately half an hour to forty-five minutes.

**STUDY PURPOSE**

Many clients at Mildmay Hospital are given ART pills that need to be taken regularly every day to keep them healthy. We developed this study to better understand the way clients at the hospital are currently taking their medication, and any adherence challenges that they may face. We are planning two studies, and depending on the results of this first study, your child may be invited to also participate in the second study if they may benefit from it. Understanding how your child takes their pills will help us develop new programs to support clients in taking their medication more frequently.

**WHY YOUR CHILD HAS BEEN APPROACHED TODAY**

Your child has been approached today because they are aged 15-17 years old inclusive and they receive their ART medication from Mildmay Uganda

**STUDY PROCEDURES**

After your child enrolls in the study (most likely today, if you decide they can participate), we will give them an electronic device called a Wisepill that measures how often they are taking their medication. The Wisepill device records the date and time each time your child opens this pillbox and sends this information to the study team through a wireless signal. Over the next 3 months, we aim to recruit a total of 1,265 participants, of which 121 will be minors. Each participant will participate in Study 1 for 3 months, and during this time, we will measure your child’s adherence using this device.

If your child decides to stop taking their ART medication before reaching the end of this study, they will still be asked to come to the hospital for their regular clinic visits. If your child wishes to leave the study, they are free to do so.

# USE OF ELECTRONIC DEVICE TO MEASURE ADHERENCE

We will give your child a Wisepill device today and ask them to store their ART medication in it while they are part of this study. We will discuss how best to fit the use of the Wisepill device into their life so that it does not affect their pill-taking. We will call them within the first week of using the device to ask whether they are having difficulties with using it. We ask that they return the Wisepill device to us at their next hospital visit which for most people is in about three months from today. If their next scheduled hospital visit is more than 3 months away, we will ask them to return to the hospital before their next scheduled visit to return the Wisepill, at the end of Study 1; if so, we will compensate them 30,000 Ugandan Shillings for transport costs and time. Please note that at any point, they can feel free to leave the study. They should not participate in the study if they do not want to, or cannot use the Wisepill device.

**RiskS AND DISCOMFORTS**

Being in this study may involve some risks and discomforts. Participating in the study requires participants to use a Wisepill device. Your child will be asked to only remove the ART medication from the device when they are about to swallow their medication. If using the Wisepill device to take their medication poses a big problem for your child, they can stop using it. Your child may not feel comfortable using the device if, for example, people who don’t know their HIV status may ask them what it is; in such cases, we have to discuss if we can find a solution that works for your child, or they may decide that they cannot participate in the study because of this issue. We may also refer them to one of the many counselors at Mildmay hospital if warranted and/or desired by your child. The counselor will follow a protocol that includes assessment of severity of the ideation, intent and means for carrying out any intent for suicide, and an appropriate plan of action (e.g., scheduling follow-up counseling, inpatient admission for psychiatric care).

**POTENTIAL BENEFITS TO PARTICIPANT AND THE SOCIETY**

We hope to gain insight into how young clients at the Mildmay Hospital are taking their medication which will allow us to develop interventions to better support youth adherence levels.

**COSTS AND COMPENSATION FOR PARTICIPATION**

There are no costs to your child for participating in the study itself. The costs related to ART and procedures that are part of your child’s usual Mildmay clinical care are their responsibility just as they were before they joined the study.

**If the parent or guardian is present on the day of recruitment for the minor, please read:** Your child will be compensated 10,000 Ugandan Shillings for their time if they decide to begin individual consenting. You will also be compensated 10,000 Ugandan Shillings for your time if you begin individual consenting.

**If the parent or guardian is not present on the day of recruitment for the minor, and the minor chose to organize a return date with their parent or guardian to Mildmay Uganda to obtain written parent or guardian consent and written minor assent, please read:** Given that we have organized you and your child to return to Mildmay Uganda for a specific study related task, you and your child will each receive 30,000 Ugandan Shillings if you decide to begin individual parent or guardian consenting and minor assenting.

**If the parent or guardian is not present on the day of recruitment for the minor, and the minor chose to organize a Study Coordinator to visit their home to obtain written parent or guardian consent and written minor assent , please read:** Given that we have organized a Study Coordinator to visit your home, you and your child will each receive 10,000 Ugandan Shillings as compensation for your time if you decide to begin individual parent or guardian consenting.

In addition, if your child participates, and completes this study, they will be compensated 30,000 Ugandan Shillings when they return to the hospital in 3 months for their final hospital visit as part of this study.

**CONFIDENTIALITY**

Your child’s information, including medical records accessed as part of the study will be kept private. Instead of their name, we will use a number to identify them. All study documents will be kept in a locked office at the Mildmay Hospital for data entry. Computer files will be protected with a password. In case of regulatory checks, National Regulatory Agencies can also access this data.

We understand that disclosure of your child’s HIV status could affect them. Therefore, only researchers will be able to see your child’s answers to consenting or survey questions. We will not share any information your child gives us, and will not share their adherence levels with their doctor; this information will only be available to the research team and will be used for research purposes only. Their name will not be used in any reports or articles we publish. We will not tell their family, their doctors, or anyone else outside the research team what they say or do during the study. However, in the case of an unforeseen event such as a fire evacuation, it is possible that your child’s privacy may be broken but we will do everything we can to prevent this. All information that identifies your child and other study related information will be stored securely by Mildmay Uganda hospital for 5 years. The data we collect will be stripped of any information that may identify your child or the hospital, and made publicly available at the end of the study.

**VOLUNTARY PARTICIPATION AND OPTION TO WITHDRAWAL**

Participation in this study is your child’s choice. If they do not want to be in the study, they can stop taking part at any time. Deciding to stop will not hurt them. They will still get the same medical care if they decide not to take part in the study.

**QUESTIONS RELATED TO THE STUDY**

If you do not understand something, or if you want more information, please ask now. If you have any questions or concerns about the research, please contact the study team below;

|  | **Name** | **Tittle** | **Contact** |
| --- | --- | --- | --- |
| 1 | Dr. Barbara Mukasa | Site Principal Investigator | 0772700816 |
| 2 | Lillian Lunkuse | Study Coordinator | 0703925522/ 0782168645 |

**WHAT ARE YOUR CHILD’S RIGHTS?**

If you or your child have questions about their rights as a research participant, please contact ethics regulatory bodies below;

| 1 | Mr. Semei Christopher Mukama | Secretary of MUREC | 0392174236 |
| --- | --- | --- | --- |
| 2 | Uganda National Council for Science and Technology |  | +256 414 705500/13. |

**DISSEMINATION OF STUDY FINDINGS**

Your child shall receive communication from the study team about the progress and findings of the study during and/or after the study findings are available to the public. We will not share their name or their client identification number in these communications.

**WRITTEN CONSENT OF PARENT GUARDIAN OF THE MINOR PARTICIPANT**

“I have read (or someone has read to me) the information provided above. I have been given an opportunity to ask questions, and all of my questions have been answered to my satisfaction.

I consent for my child to participate in the research as described above.

YES (If YES, Continue with signing the consent form. If NO, thank them and stop the consenting process). NO

Guardian/ parent has been given a copy of the form YES NO

Name of Child________________________________________________________________

Name of Parent or Guardian ____________________________________________________

Signature / thumbprint of Parent or Guardian_______________________________________

Date________________________________________

Name of Parent or Guardian (written by a witness if the participant is unable to read and write)

Name of Parent or Guardian ____________________________________________________

Date _______________________________________________

Name of witness (if the Parent or Guardian is unable to read and write)________________________________________________________________

Signature of Witness__________________________________________________

Date___________________________________________________

**SIGNATURE OF STUDY INTERVIEWER**

I have explained the research to the participant’s parent or guardian and answered all of his/her questions. I believe that he/she understands the information described in this document and freely consents for their child to participate.

Name of Study Interviewer______________________________________________________

Signature of Study Interviewer___________________________________________________

Date_________________________________________________

### **2.2 Study 1 Minor Assent form**

Study ID………… Clinic ID……………………………..

**Assent Form for Minors Study 1 (15-17 years old)**

**“Goals for Adherence with Low-cost Incentives (GOALS)”**

**Lead Principal Investigator:**

Dr. Sebastian Linnemayr, PhD

Institution of affiliation: RAND Corporation

Address: 1776 Main Street, Santa Monica, CA 90407-2138, US

Phone contact: +1 310 393 0411 ext 6734

Email: slinnema@rand.org

**Principal Investigator:**

Dr. Crystal Haijing Huang, PhD

Institution of affiliation: IDinsight

Address: 44 Tehama Street, San Francisco, CA 94105, US

Phone contact: +1 202 436 1411

Email: crystal.huang@idinsight.org

**Site Principal investigator:**

Dr. Barbara Mukasa

Institution of affiliation: Mildmay Uganda

Address: P.O. Box 24985, Kampala; Uganda

Phone contact: +256 312 210 200 or +256 772 700 816

Email: barbara.mukasa@mildmay.or.ug

**Sponsor:** National Institute of Child Health and Human Development (NICHD), USA

**Collaborating Institutions:** IDinsight, The RAND Corporation, Mildmay Uganda

**INTRODUCTION**

Hello, my name is [insert name of the Study Coordinator]. I work at Mildmay Hospital that together with the RAND Corporation, and IDinsight, is conducting a study at Mildmay Uganda. The purpose of this study is to understand antiretroviral therapy (ART) medication adherence among young clients at Mildmay Hospital. This study has been approved by an accredited Ugandan-based Research Ethics Committee, the Mildmay Uganda Research Ethics Committee.

Before you decide whether or not you want to participate in this study, I would like to explain what the study is about, how it may help you, any risks to you, and what is expected of you. After I discuss the details of the study with you, you will be asked to give written assent if you decide that you want to be a part of the study. This process will take approximately half an hour to forty-five minutes.

**STUDY PURPOSE**

Many clients at the Mildmay Hospital are given ART pills. These medications need to be taken regularly every day to keep you healthy. We developed this study to better understand the way clients at Mildmay like yourself are currently taking their medication, and any adherence challenges that they may face. We are planning two studies, and depending on the results of this first study, you may be invited to also participate in the second study if you would benefit from it. Understanding how you take your pills will help us develop new programs to support clients in taking their medication more frequently.

**WHY YOU HAVE BEEN APPROACHED TODAY**

You have been approached today because you are aged 15-17 years old inclusive and you receive your ART medication from Mildmay Uganda

**STUDY PROCEDURES**

After you enroll in the study (most likely today, if you decide to participate), we will give you an electronic device called Wisepill that measures how often you are taking your medication. The Wisepill device records the date and time each time you open this pillbox and sends this information to the study team through a wireless signal. Over the next 3 months, we aim to recruit a total of 1,265 participants, of which 121 will be minors. Each participant will participate in Study 1 for 3 months, and during this time, we will measure your adherence using this device.

If you decide to stop taking your ART medication before reaching the end of this study, you will still be asked to come to the hospital for your regular clinic visits. If you wish to leave the study, you are free to do so.

# USE OF ELECTRONIC DEVICE TO MEASURE ADHERENCE

We will give you a Wisepill device today and ask you to store your ART medication in it while you are part of this study. We will discuss how best to fit the use of the Wisepill device into your life so that it does not affect your pill-taking. We will call your child within the first week of using the device to ask whether you are having difficulties with using it. We ask that you return the Wisepill device to us at your next hospital visit which for most people is in about three months from today. If your next scheduled hospital visit is more than 3 months away, we will ask you to return to the hospital before your next scheduled visit to return the Wisepill, at the end of Study 1; if so, we will compensate you 30,000 Ugandan Shillings for transport costs and time. Please note that at any point, you can feel free to leave the study. You should not participate in the study if you do not want to, or cannot use the Wisepill device.

**RiskS AND DISCOMFORTS**

Being in this study may involve some risks and discomforts. Participating in the study requires participants to use a Wisepill device. You will be asked to only remove the ART medication from the device when you are about to swallow the medication. If using the Wisepill device to take your medication poses a big problem for you, you can stop using it. You may not feel comfortable using the device if, for example, people who don’t know your HIV status ask you what it is; in such cases, we have to discuss if we can find a solution that works for you, or you may decide that you cannot participate in the study because of this issue. We may also refer you to one of the many counselors at Mildmay hospital if warranted and/or desired by yourself. The counselor will follow a protocol that includes assessment of severity of the ideation, intent and means for carrying out any intent for suicide, and an appropriate plan of action (e.g., scheduling follow-up counseling, inpatient admission for psychiatric care).

**POTENTIAL BENEFITS TO PARTICIPANT AND THE SOCIETY**

We hope to gain insight into how young clients at the Mildmay Hospital are taking their medication which will allow us to develop interventions to better support youth adherence levels.

**COSTS AND COMPENSATION FOR PARTICIPATION**

There are no costs to you for participating in the study itself. The costs related to ART and procedures that are part of your usual Mildmay clinical care are your responsibility just as they were before you joined the study.

**If the parent or guardian is present on the day of recruitment for the minor, please read:** If you decide to begin the individual assenting process today you will be compensated 10,000 Ugandan Shillings for your time. Your parent or guardian will complete the consenting process today on your behalf and they will also be compensated 10,000 Ugandan Shillings for their time.

**If the parent or guardian is not present on the day of recruitment for the minor, and the minor chose to organize a return date with their parent or guardian to Mildmay Uganda to obtain written parent or guardian consent and written minor assent, please read:** Given that we have organized you and your parent or guardian to return to Mildmay Uganda for a specific study related task, you and your parent or guardian will each receive 30,000 Ugandan Shillings upon beginning individual minor assent and parent or guardian consent.

**If the parent or guardian is not present on the day of recruitment for the minor, and the minor chose to organize a Study Coordinator to visit their home to obtain written parent or guardian consent and written minor assent , please read:** Given that we have organized a Study Coordinator to visit your home, you and your parent or guardian will each receive 10,000 Ugandan Shillings upon beginning individual minor assent and parent or guardian consent.

In addition, if you participate, and complete this study, you will be compensated 30,000 Ugandan Shillings when you return to the hospital in 3 months for your final hospital visit as part of this study.

**CONFIDENTIALITY**

Your information, including medical records accessed as part of the study will be kept private. Instead of your name, we will use a number to identify you. All study documents will be kept in a locked office at the Mildmay Hospital for data entry. Computer files will be protected with a password. In case of regulatory checks, National Regulatory Agencies can also access this data.

We understand that disclosure of your HIV status could could affect you. Therefore, only researchers will be able to see your answers to consenting or survey questions. We will not share any information you give us, and will not share your adherence levels with your doctor; this information will only be available to the research team and will be used for research purposes only. Your name will not be used in any reports or articles we publish. We will not tell your family, your doctors, or anyone else outside the research team what you say or do during the study. However, in the case of an unforeseen event such as a fire evacuation, it is possible that your privacy may be broken but we will do everything we can to prevent this. All information that identifies you and other study related information will be stored securely by Mildmay Uganda hospital for 5 years. The data we collect will be stripped of any information that may identify you or the clinic, and made publicly available at the end of the study.

**VOLUNTARY PARTICIPATION AND OPTION TO WITHDRAWAL**

Participation in this study is your choice. If you do not want to be in the study, you can stop taking part at any time. Deciding to stop will not hurt you. You will still get the same medical care if you decide not to take part in the study.

**QUESTIONS RELATED TO THE STUDY**

If you do not understand something, or if you want more information, please ask now. If you do not understand something, or if you want more information, please ask now. If you have any questions or concerns about the research, please contact the study team below;

|  | **Name** | **Tittle** | **Contact** |
| --- | --- | --- | --- |
| 1 | Dr. Barbara Mukasa | Site Principal Investigator | 0772700816 |
| 2 | Lillian Lunkuse | Study Coordinator | 0703925522/ 0782168645 |

**WHAT ARE YOUR CHILD’S RIGHTS?**

If you or your child have questions about their rights as a research participant, please contact ethics regulatory bodies below;

| 1 | Mr. Semei Christopher Mukama | Secretary of MUREC | 0392174236 |
| --- | --- | --- | --- |
| 2 | Uganda National Council for Science and Technology |  | +256 414 705500/13. |

**DISSEMINATION OF STUDY FINDINGS**

You shall receive communication from the study team about the progress and findings of the study during and/or after the study findings are available to the public. We will not share your name or your client identification number in these communications.

**WRITTEN ASSENT OF RESEARCH PARTICIPANT**

“I have read (or someone has read to me) the information provided above. I have been given an opportunity to ask questions, and all of my questions have been answered to my satisfaction.

I assent to participate in the research as described above.

YES (If YES, Continue with signing the assent form. If NO, thank the participant and stop the consenting process). NO

I have chosen to take a copy of this assent form

YES NO

Name of Participant_______________________________________________________________

Signature / thumbprint of Participant_________________________________________________

Date________________________________________

Name of participant (written by a witness if the participant is unable to read and write)

Name of Participant __________________________________________________________

Date _______________________________________________

Name of Witness (if the participant is unable to read and write)______________________________________________________________________

Signature of Witness________________________________________________

Date___________________________________________________________

**SIGNATURE OF STUDY INTERVIEWER**

I have explained the research to the participant and answered all of his/her questions. I believe that he/she understands the information described in this document and freely assents to participate.

Name of Study Intervier________________________________________________________________

Signature of Study Interviewer__________________________________________

Date__________________________________________________

### **2.3 Study 1 Non-minor consent form**

Study ID……………………… ClinicID……………………………..

**Consent Form for Non-Minors Study 1 (18 years old or above)**

**“Goals for Adherence with Low-cost Incentives (GOALS)”**

**Lead Principal Investigator:**

Dr. Sebastian Linnemayr, PhD

Institution of affiliation: RAND Corporation

Address: 1776 Main Street, Santa Monica, CA 90407-2138, US

Phone contact: +1 310 393 0411 ext 6734

Email: slinnema@rand.org

**Principal Investigator:**

Dr. Crystal Haijing Huang, PhD

Institution of affiliation: IDinsight

Address: 44 Tehama Street, San Francisco, CA 94105, US

Phone contact: +1 202 436 1411

Email: crystal.huang@idinsight.org

**Site Principal investigator:**

Dr. Barbara Mukasa

Institution of affiliation: Mildmay Uganda

Address: P.O. Box 24985, Kampala; Uganda

Phone contact: +256 312 210 200 or +256 772 700 816

Email: barbara.mukasa@mildmay.or.ug

**Sponsor:** National Institute of Child Health and Human Development (NICHD), USA

**Collaborating Institutions:** IDinsight, The RAND Corporation, Mildmay Uganda

**INTRODUCTION**

Hello, my name is [insert name of the Study Coordinator]. I work at Mildmay Hospital that together with the RAND Corporation, and IDinsight, is conducting a study at Mildmay Uganda. The purpose of this study is to understand antiretroviral therapy (ART) medication adherence among young clients at Mildmay Hospital. This study has been approved by an accredited Ugandan-based Research Ethics Committee, the Mildmay Uganda Research Ethics Committee.

Before you decide whether or not you want to participate in this study, I would like to explain what the study is about, how it may help you, any risks to you, and what is expected of you. After I discuss the details of the study with you, I will ask you if you want to be part of the study and if yes, you will be asked to sign an Informed Consent form. This process will take approximately half an hour to forty minutes.

**STUDY PURPOSE**

Many clients at Mildmay Hospital are given ART pills that need to be taken regularly every day to keep them healthy. We developed this study to better understand the way clients at the hospital are currently taking their medication, and any adherence challenges that they may face. We are planning two studies, and depending on the results of this first study, you may be invited to also participate in the second study if you would benefit from it. Understanding how you take your pills will help us develop new programs to support clients in taking their medication more frequently.

**WHY YOU HAVE BEEN APPROACHED TODAY**

You have been approached today because you are aged 15-30 years old inclusive and you receive your ART medication from Mildmay Uganda

**STUDY PROCEDURES**

After you enroll in the study (most likely today, if you decide to participate), we will give you an electronic device called Wisepill that measures how often you take your medication. The Wisepill device records the date and time each time you open this pillbox and sends this information to the study team through a wireless signal. Over the next 3 months, we aim to recruit a total of 1,265 participants, of which 121 will be minors. Each participant will participate in Study 1 for 3 months, and during this time, we will measure your adherence using this device.

If you decide to stop taking your ART medication before reaching the end of this study, you will still be asked to come to the hospital for your regular clinic visits. If you wish to leave the study, you are free to do so.

# USE OF ELECTRONIC DEVICE TO MEASURE ADHERENCE

We will give you a Wisepill device today and ask you to store your ART medication in it while you are part of this study. We will discuss how best to fit the use of the Wisepill device into your life so that it does not affect your pill-taking. We will call you within the first week of using the device to ask whether you are having difficulties with using it. We ask that you return the Wisepill device to us at your next hospital visit which for most people is in about three months from today. If your next scheduled hospital visit is more than 3 months away, we will ask you to return to the hospital before your next scheduled visit to return the Wisepill, at the end of Study 1; if so, we will compensate you 30,000 Ugandan Shillings for transport costs and time. Please note that at any point, you can feel free to leave the study. You should not participate in the study if you do not want to, or cannot use the Wisepill device.

**RiskS AND DISCOMFORTS**

Being in this study may involve some risks and discomforts. Participating in the study requires participants to use a Wisepill device. You will be asked to only remove the ART medication from the device when you are about to swallow your medication. If using the Wisepill device to take your medication poses a big problem for you, you can stop using it. You may not feel comfortable using the device if, for example, people who don’t know your HIV status ask you what it is; in such cases, we have to discuss if we can find a solution that works for you, or you may decide that you cannot participate in the study because of this issue. We may also refer you to one of the many counselors on staff at the Mildmay hospital if warranted and/or desired by yourself. The counselor will follow a protocol that includes assessment of severity of the ideation, intent and means for carrying out any intent for suicide, and an appropriate plan of action (e.g., scheduling follow-up counseling, inpatient admission for psychiatric care).

**POTENTIAL BENEFITS TO PARTICIPANT AND THE SOCIETY**

We hope to gain insight into how young clients at the Mildmay Hospital are taking their medication which will allow us to develop interventions to better support youth adherence levels.

**COSTS AND COMPENSATION FOR PARTICIPATION**

There are no costs to you for participating in the study itself. The costs related to ART and procedures that are part of your usual Mildmay Uganda clinical care are your responsibility just as they were before you joined the study. However, if you decide to begin individual consenting today, you will be compensated 10,000 Ugandan Shillings for your time. In addition, if you participate, and complete this study, you will be compensated 30,000 Ugandan Shillings when you return to the hospital in 3 months for your final hospital visit as part of this study.

**CONFIDENTIALITY**

Your information, including medical records accessed as part of the study will be kept private. Instead of your name, we will use a number to identify you. All study documents will be kept in a locked office at the Mildmay Hospital for data entry. Computer files will be protected with a password. In case of regulatory checks, National Regulatory Agencies can also access this data.

We understand that disclosure of your HIV status could affect you. Therefore, only researchers will be able to see your answers to consenting or survey questions. We will not share any information you give us, and will not share your adherence levels with your doctor; this information will only be available to the research team and will be used for research purposes only. Your name will not be used in any reports or articles we publish. We will not tell your family, your doctors, or anyone else outside the research team what you say or do during the study. However, in the case of an unforeseen event such as a fire evacuation, it is possible that your privacy may be broken but we will do everything we can to prevent this. All information that identifies you and other study related information will be stored securely by Mildmay Uganda hospital for 5 years. The data we collect will be stripped of any information that may identify you or the hospital, and made publicly available at the end of the study.

**VOLUNTARY PARTICIPATION AND OPTION TO WITHDRAWAL**

Participation in this study is your choice. If you do not want to be in the study, you can stop taking part at any time. Deciding to stop will not hurt you. You will still get the same medical care if you decide not to take part in the study.

**QUESTIONS RELATED TO THE STUDY**

If you do not understand something, or if you want more information, please ask now. If you do not understand something, or if you want more information, please ask now. If you have any questions or concerns about the research, please contact the study team below;

|  | **Name** | **Tittle** | **Contact** |
| --- | --- | --- | --- |
| 1 | Dr. Barbara Mukasa | Site Principal Investigator | 0772700816 |
| 2 | Lillian Lunkuse | Study Coordinator | 0703925522/ 0782168645 |

**WHAT ARE YOUR CHILD’S RIGHTS?**

If you or your child have questions about their rights as a research participant, please contact ethics regulatory bodies below;

| 1 | Mr. Semei Christopher Mukama | Secretary of MUREC | 0392174236 |
| --- | --- | --- | --- |
| 2 | Uganda National Council for Science and Technology |  | +256 414 705500/13. |

**DISSEMINATION OF STUDY FINDINGS**

You shall receive communication from the study team about the progress and findings of the study during and/or after the study findings are available to the public. We will not share your name or your client identification number in these communications.

**WRITTEN CONSENT OF RESEARCH PARTICIPANT**

“I have read (or someone has read to me) the information provided above. I have been given an opportunity to ask questions, and all of my questions have been answered to my satisfaction.

I consent to participate in the research as described above.

YES (If YES, Continue with signing the consent form, if NO, thank the participant and stop the consenting process). NO
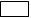


I have chosen to take a copy of this consent form

YES NO

Name of Participant_______________________________________________________________

Signature / thumbprint of Participant________________________________________________

Date_______________________________________________

Name of participant (written by a witness if the participant is unable to read and write)

Name of Participant _____________________________________________________

Date _______________________________________________

Name of Witness (if the participant is unable to read and write)__________________________________________________________

Signature of Witness_________________________________________

Date____________________________________________

**SIGNATURE OF STUDY INTERVIEWER**

I have explained the research to the participant and answered all of his/her questions. I believe that he/she understands the information described in this document and freely consents to participate.

Name of Study Interviewer________________________________________________________

Signature of Study Interviewer_______________________________________________

Date___________________________________________

### **2.4 Study 2 Minor Assent form**

**Assent form for Minors Study 2**

**“Goals for Adherence with Low-cost Incentives (GOALS)”**

**Lead Principal Investigator:**

Dr. Sebastian Linnemayr, PhD

Institution of affiliation: RAND Corporation

Address: 1776 Main Street, Santa Monica, CA 90407-2138, US

Phone contact: +1 310 393 0411 ext 6734

Email: slinnema@rand.org

**Principal Investigator:**

Dr. Crystal Haijing Huang, PhD

Institution of affiliation: IDinsight

Address: 44 Tehama Street, San Francisco, CA 94105, US

Phone contact: +1 202 436 1411

Email: crystal.huang@idinsight.org

**Site Principal investigator:**

Dr. Barbara Mukasa

Institution of affiliation: Mildmay Uganda

Address: P.O. Box 24985, Kampala; Uganda

Phone contact: +256 312 210 200 or +256 772 700 816

Email: barbara.mukasa@mildmay.or.ug

**Sponsor:** National Institute of Child Health and Human Development (NICHD), USA

**Collaborating Institutions:** IDinsight, The RAND Corporation, Mildmay Uganda

**INTRODUCTION**

Hello, my name is [insert name of the Study Coordinator]. I work at Mildmay Uganda that in collaboration with the RAND Corporation, and IDinsight, is conducting a study at Mildmay Uganda. The purpose of this study is to understand antiretroviral therapy (ART) medication adherence among young clients at Mildmay Hospital. This study has been approved by an accredited Ugandan-based Research Ethics Committee, the Mildmay Uganda Research Ethics Committee

We would like to thank you for participating in the previous study which measured your adherence using a Wisepill device. We would like to invite you to participate in a new study, which aims to help clients such as yourself take their HIV medication. Before you decide whether or not you want to participate in this new study, we would like to explain why we are doing it, how it may help you, any risks to you, and what is expected of you. After I talk about the details of the study with you, I will ask you if you want to be part of the study and if yes, you will be asked to give verbal assent.

**STUDY PURPOSE**

Many young people living with HIV have trouble taking their HIV medication every day to receive the most benefit. Therefore, we developed this study called “**Go**als for **A**dherence with **L**ow-cost Incentive**s**” (GOALS), which can help participants such as yourself improve your motivation for taking ART medication, and ultimately reach and maintain a high adherence level. If participants maintain a certain level of adherence, they will be eligible to win prizes between 500 and 10,000 Ugandan Shillings of mobile airtime approximately every 3 months during the first 24 months of the study. Adherence will be measured with the Wisepill device. Additionally, participants may be able to win a larger prize at the end of 12 and 24 months, worth up to 20,000 Ugandan Shillings airtime each.

**STUDY PROCEDURES**

If you decide to participate, you will continue to use the same Wisepill device. You will also be requested to complete a 30-minute survey today if you decide to participate. This questionnaire will be repeated every six months and will include questions about your background, medical history, symptoms, and your attitude to pill taking. Every time you come for a scheduled hospital visit, we will also ask you to come to this research office. Whenever possible, the study activities will be scheduled on days on which you have a visit already scheduled at Mildmay Hospital so you don’t have to come to the hospital for study-related reasons only.

A total of 628 HIV participants from the Mildmay Hospital who have been receiving ART treatment for at least three months prior to the intervention will take part in the study. You will be assigned to one of the following four groups and will have an equal (i.e., one in four) chance of being in either of the four groups as the assignment is randomly allocated by a computer. You will not be able to choose the group you want to be in.

**Participants in Group 1:** For a Group 1 participant **the study coordinator will choose a target based on your current adherence and gradually increase the target every 3 months**. The goal is to achieve a 90% level of adherence by the end of year one. For example, if your initial adherence level is 70%, your adherence target to be eligible to win a prize after 3 months may be 75%. This will continue until you reach 90%.

**Participants in Group 2:** Group 2 participants can **choose their own adherence goal subject to a minimum threshold every 3 months.** The goal is to achieve a 90% level of adherence by the end of year one. For example, if your initial adherence level is 70%, **you** yourself may set your target to be 75%. This will continue until you reach 90%.

**Participants in Group 3:** For Group 3 participants to be eligible for a prize drawing, **you must reach a target adherence level of 90% every 3 months.**

Every 3 months, we will check your adherence and conduct a prize drawing if you reached your target. If you have a hospital visit scheduled when you are due for a prize drawing (every 3 months), we ask that you come to this research office. We will do the prize drawing in person if you reached your target. If you do not have a hospital visit scheduled when you are due for a prize drawing, we will send you an SMS message notifying you of your adherence performance and whether you have achieved your target. If you reached the target, the computer will determine the amount you won; we will inform you of the amount and send you your prize in the form of mobile airtime. If you are in Group 2, we will ask you to text us what you would like your next target to be. All participants will receive a weekly text message with a motivational message to keep working towards your target.

No personal, private or sensitive information will be included in any text message you receive from us. The text message will not include words such as ‘HIV’ or ‘ART medication’, and will not share or imply your HIV status. For example, a text message could say *“Hello, this is GOALS. Congratulations! You are at 70% and your target was 85%. The computer determined that your prize is 10,000 UGX. We will now send you this as Airtime”.*

For the first 12 months, participants in Groups 1, 2, and 3 can win a prize between 500 and 10,000 Ugandan Shillings in mobile airtime every 3 months if they reach the pre-determined target.

After 12 months, if you achieve 90% adherence, we will ask you to maintain this level in order to be eligible for a prize drawing approximately every 3 months. If you do not achieve 90% adherence, you will be given another 6 months to improve to 90%. There will be a larger prize drawing if you achieve 90% adherence at the end of month 12 or month 18, depending on the category of adherence you fall under. You will be entered into a prize draw where you may win up to 20,000 Ugandan Shillings of airtime.

Similarly, a larger prize draw will happen if you achieve viral suppression by the end of month 24 or month 30, depending on the category of adherence you fall under after month 12.

After these 30 months, there will be no more prize draws but we will keep measuring your adherence for another 6 months to see how you take your medication during this time.

**Participants in Group 4:** **You will receive the usual standard of care** as offered to all clients at Mildmay Hospital. You will also receive weekly text messages, but there will not be prize drawings in this group. We will ask you to use your Wisepill device for 36 months, and come to the research office each time you come to Mildmay for a hospital visit. We will also give you 30,000 UGX for your transport and time on the 6^th^, 12^th^, 18^th^ and 24^th^ month when you come back to complete adherence surveys.

I can’t tell you which group you will be in until you tell me whether you agree to continue in the study.

**SCHEDULE OF FOLLOW UP VISITS**

No matter which group you are assigned to, you will continue to come to Mildmay Hospital for your HIV care on your regularly scheduled days. If you decide to stop taking your ART medication before reaching the end of the study, you will be asked to still come to the hospital for all the remaining study visits. If you wish to leave the study and not continue with the scheduled study visits, you are free to do so.

# USE OF ELECTRONIC DEVICE TO MEASURE ADHERENCE

Over the course of the study, you will be asked to use the Wisepill device and bring it to each next hospital visit. Please note that at any point, you may choose not to continue participation in the study. You should not participate in the study if you do not want to or cannot use the Wisepill device. In intervention Groups 1, 2 and 3, the prize drawings which happen approximately every 3 months are based on achieving your specific adherence level measured by the Wisepill device.

**Risk AND DISCOMFORTS**

Being in this study may involve some risks and discomforts. Participating in the study requires participants to use the electronic Wisepill device. You will be asked to only remove the ART medication from the device when you are about to swallow the medication. If you don’t take the pills the way the doctor tells you to, this may reduce the effectiveness of the medication and increase the risk of developing drug resistance. If using the electronic device to take your medication poses a big problem for you, you can stop using it. You may not feel comfortable using the device if, for example, people who don’t know your HIV status ask you what it is; in such cases, we have to discuss if we can find a solution that works for you, or we may decide that you cannot participate in the study because of this issue.

If you are in Group 1, 2 or 3, you may be able to participate in prize drawings; you may feel pressure to come to the hospital and take your medications or feel stress when you do not win a prize because you were not eligible for the drawing. Similarly, you may feel upset because of things discussed in the questionnaire.

We may also refer you to one of the many counselors on staff at the Mildmay hospital if warranted and/or desired by yourself. The counselor will follow a protocol that includes assessment of severity of the ideation, intent and means for carrying out any intent for suicide, and an appropriate plan of action (e.g., scheduling follow-up counseling, inpatient admission for psychiatric care).

**POTENTIAL BENEFITS TO PARTICIPANT AND THE SOCIETY**

The study helps us better understand how clients at the Mildmay Hospital are taking their medication which will allow us to develop the interventions to better support youth adherence levels.

**COSTS AND COMPESTAION FOR PARTICIPATION**

There are no costs to you for participating in the study itself. The costs related to ART and procedures that are part of your usual Mildmay clinical care are your responsibility just as they were before you joined the study.

If you are assigned to Group 1, 2 or 3 and if you achieve the pre-determined adherence level target **approximately every** **3 months within the first 24 months of the study period**, you will be invited to participate in a prize drawing where you can win between 500 and 10,000 Ugandan Shillings of mobile airtime; the value of the prize is not guaranteed as the prize is drawn by chance.

In addition, if you either achieve 90% adherence by the end of year 1, or you achieve viral suppression by the end of year 2, or both, you will be entered into an end of year prize draw where you may up to an amount of 20,000 Ugandan Shillings of airtime each.

We will also compensate you 30,000 UGX after completing the periodic adherence surveys at months 6, 12, 18 and 24.

**CONFIDENTIALITY**

Your answers, including medical records accessed as part of the study will be kept private. Instead of your name, we will use a number to identify you. All study documents will be kept in a locked office at the Mildmay Hospital for data entry. Computer files will be protected with a password.

We understand that disclosure of your HIV status could be embarrassing or could cause damage to your reputation. Therefore, only researchers will be able to see your answers. We will not share any information you give us, and will not share your adherence levels with your doctor; this information will only be available to the research team and will be used for research purposes only. Your name will not be used in any reports or articles we publish. We will not tell your family, your doctors, or anyone else outside the research team what you say or do during the study. However, it is possible that your privacy will be broken. We will do everything we can to prevent this. When we talk about the study in reports or articles, we will hide your name and other things about who you are. All information that identifies you will be destroyed at the end of the study. The data we collect will be stripped of any information that may identify you or the hospital, and made publicly available at the end of the study.

**VOLUNTARY PARTICIPATION AND OPTION TO WITHDRAWAL**

Participation in this study is your choice. If you do not want to be in the study, you can stop taking part at any time. Deciding to stop will not hurt you. You will still get the same medical care if you decide not to take part in the study.

**QUESTIONS RELATED TO THE STUDY**

If you do not understand something, or if you want more information, please ask now. If you have any questions or concerns about the research, please contact Dr. Barbara Mukasa, Site Principal Investigator, at 0772700816 or Mr. Peter Wabukala, the Study Coordinator, at 0785823296

**RIGHTS OF RESEARCH PARTICIPANTS**

If you have questions about your rights as a research participant, please contact Semei Christopher Mukama, Secretary of the MUREC, at 0392174236 or Uganda National Council for Science and Technology at +256 414 705500/13

**DISSEMINATION OF STUDY FINDINGS**

You shall receive communication from the study team about the progress and findings of the study during and/or after the study findings are available to the public. We will not share your name or your client identification number in these communications.

**WRITTEN ASSENT OF RESEARCH PARTICIPANT**

“I have read (or someone has read to me) the information provided above. I have been given an opportunity to ask questions, and all of my questions have been answered to my satisfaction.

I have chosen to take a copy of this assent form

YES (If YES, Continue to with signing the assent form). NO

I assent to participate in the research as described above.

YES (If YES, Continue with signing the assent form). NO

Name of Participant ________________________________________

Signature / thumbprint of Participant ___________________________

Date _______________________________________________

Name of Witness (if unable to read and write) _________________________________

Signature of Witness ______________________________

Date ___________________________________________

**SIGNATURE OF STUDY INTERVIEWER**

I have explained the research to the participant and answered all of his/her questions. I believe that he/she understands the information described in this document and freely assents to participate.

Name of Study Interviewer ________________________________________

Signature of Study Interviewer ___________________________________

Date ___________________________________________________________

### **2.5 Study 2 Guardian Consent form**

**Consent form for Guardians Study 2**

**“Goals for Adherence with Low-cost Incentives (GOALS)”**

**Lead Principal Investigator:**

Dr. Sebastian Linnemayr, PhD

Institution of affiliation: RAND Corporation

Address: 1776 Main Street, Santa Monica, CA 90407-2138, US

Phone contact: +1 310 393 0411 ext 6734

Email: slinnema@rand.org

**Principal Investigator:**

Dr. Crystal Haijing Huang, PhD

Institution of affiliation: IDinsight

Address: 44 Tehama Street, San Francisco, CA 94105, US

Phone contact: +1 202 436 1411

Email: crystal.huang@idinsight.org

**Site Principal investigator:**

Dr. Barbara Mukasa

Institution of affiliation: Mildmay Uganda

Address: P.O. Box 24985, Kampala; Uganda

Phone contact: +256 312 210 200 or +256 772 700 816

Email: barbara.mukasa@mildmay.or.ug

**Sponsor:** National Institute of Child Health and Human Development (NICHD), USA

**Collaborating Institutions:** IDinsight, The RAND Corporation, Mildmay Uganda

**INTRODUCTION**

Hello, my name is [insert name of the Study Coordinator]. I work at Mildmay Uganda that in collaboration with the RAND Corporation, and IDinsight, is conducting a study at Mildmay Uganda. The purpose of this study is to understand antiretroviral therapy (ART) medication adherence among young clients at Mildmay Hospital. This study has been approved by an accredited Ugandan-based Research Ethics Committee, the Mildmay Uganda Research Ethics Committee

We would like to thank your child for participating in the previous study which measured their adherence using a Wisepill device. We would like to invite your child to participate in a new study, which aims to help clients such as your child take their HIV medication. Before you decide whether or not your child will participate in this study, we would like to explain why we are doing it, how it may help your child, any risks to your child, and what is expected of your child. After I talk about the details of the study with you, I will ask you if you want your child to be part of the study and if yes, you will be asked to sign this Informed Consent form on their behalf.

**STUDY PURPOSE**

Many young people living with HIV have trouble taking their HIV medication every day to receive the most benefit. Therefore, we developed this study called “**Go**als for **A**dherence with **L**ow-cost Incentive**s**” (GOALS), which can help participants such as your child improve their motivation for taking ART medication, and ultimately reach and maintain a high adherence level. If your child maintains a certain level of adherence, they will be eligible to win prizes between 500 and 10,000 Ugandan Shillings of mobile airtime approximately every 3 months during the first 24 months of the study. Adherence will be measured with a Wisepill device. Additionally, your child may be able to win a larger prize at the end of 12 and 24 months, worth up to 20,000 Ugandan Shillings airtime each.

**STUDY PROCEDURES**

If your child decides to participate, they will continue to use the same Wisepill device. Your child will also be requested to complete a 30-minute survey today if you decide that they will participate. This questionnaire will be repeated every six months and will include questions about their background, medical history, symptoms, and their attitude to pill taking. Every time your child comes for a scheduled hospital visit, we will also ask them to come to this research office. Whenever possible, the study activities will be scheduled on days on which your child has a visit already scheduled at Mildmay Hospital so they don’t have to come to the hospital for study-related reasons only.

A total of 628 HIV positive participants from the Mildmay Hospital who have been receiving ART treatment for at least three months prior to the intervention will take part in the study. Your child will be assigned to one of the following four groups and will have an equal (i.e., one in four) chance of being in either of the four groups as the assignment is randomly allocated by a computer. Your child will not be able to choose the group they want to be in.

**Participants in Group 1:** For a Group 1 participant **the study coordinator will choose a target based on your child’s current adherence and gradually increase the target every 3 months**. The goal is to achieve a 90% level of adherence by the end of year one. For example, if your child’s initial adherence level is 70%, their adherence target to be eligible to win a prize after 3 months may be 75%. This will continue until they reach 90%.

**Participants in Group 2** Group 2 participants can **choose their own adherence goal subject to a minimum threshold every 3 months.** The goal is to achieve a 90% level of adherence by the end of year one. For example, if your child’s initial adherence level is 70%, **your child** may set their target to be 75%. This will continue until they reach 90%.

**Participants in Group 3:** For Group 3 participants to be eligible for a prize drawing, **your child** **must reach a target adherence level of 90% every 3 months.**

Every 3 months, we will check your child’s adherence and conduct a prize drawing if they reached their target. If your child has a hospital visit scheduled when they are due for a prize drawing (every 3 months), we ask that they come to this research office. We will do the prize drawing in person if your child reached their target. If they do not have a hospital visit scheduled when they are due for a prize drawing, we will send them an SMS message notifying them of their adherence performance and whether they have achieved their target. If they reached the target, the computer will determine the amount they won; we will inform your child of the amount and send them their prize in the form of mobile airtime. If they are in Group 2, we will ask them to text us what they would like their next target to be. All participants will receive a weekly text message with a motivational message.

No personal, private or sensitive information will be included in any text message your child receives from us. The text message will not include words such as ‘HIV’ or ‘ART medication’, and will not share or imply their HIV status. For example, a text message could say *“Hello, this is GOALS. Congratulations! You are at 70% and your target was 85%. The computer determined that your prize is 10,000 UGX. We will now send you this as Airtime”.*

For the first 12 months, participants in Groups 1, 2, and 3 can win a prize at each hospital visit between 500 and 10,000 Ugandan Shillings in mobile airtime if they reach the pre-determined targets.

After 12 months, if you achieve 90% adherence, we will ask you to maintain this level in order to be eligible for a prize drawing approximately every 3 months. If you do not achieve 90% adherence, you will be given another 6 months to improve to 90%. There will be a larger prize drawing if you achieve 90% adherence at the end of month 12 or month 18, depending on the category of adherence you fall under. You will be entered into a prize draw where you may win up to 20,000 Ugandan Shillings of airtime.

Similarly, a larger prize draw will happen if you achieve viral suppression by the end of month 24 or month 30, depending on the category of adherence you fall under after month 12.

After these 30 months, there will be no more prize draws but we will keep measuring your adherence for another 6 months to see how you take your medication during this time.

**Participants in Group 4:** **Your child will receive the usual standard of care** as offered to all clients at Mildmay Hospital. Your child will also receive weekly text messages, but there will not be prize drawings in this group. We will ask your child to use their Wisepill device for 36 months, and come to the research office each time they come to Mildmay for a clinic visit. We will also give your child 30,000 UGX for transport and their time on the 6^th^, 12^th^, 18^th^ and 24^th^ month when they come back to complete adherence surveys. I can’t tell you which group your child will be in until you tell me whether you agree for your child to continue in the study.

**SCHEDULE OF FOLLOW UP VISITS**

No matter which group your child is assigned to, they will continue to come to Mildmay Hospital for their HIV care on their regularly scheduled days. If they decide to stop taking their ART medication before reaching the end of the study, they will be asked to still come to the hospital for all the remaining study visits. If they wish to leave the study and not continue with the scheduled study visits, they are free to do so.

# USE OF ELECTRONIC DEVICE TO MEASURE ADHERENCE

Over the course of the study, your child will be asked to use the Wisepill device and bring it to each hospital visit. Please note that at any point, they may choose not to continue participation in the study. They should not participate in the study if they do not want to or cannot use the Wisepill device. In intervention Group 1, 2 and 3, the prize drawings which happen approximately every 3 months are based on your child achieving their specific adherence level measured by the Wisepill device.

**Risk AND DISCOMFORTS**

Being in this study may involve some risks and discomforts. Participating in the study requires participants to use the electronic Wisepill device. Your child will be asked to only remove the ART medication from the device when they are about to swallow the medication. If they don’t take the pills the way the doctor tells them to, this may reduce the effectiveness of the medication and increase the risk of developing drug resistance. If using the electronic device to take their medication poses a big problem for them, they can stop using it. Your child may not feel comfortable using the device if, for example, people who don’t know their HIV status ask them what it is; in such cases, we have to discuss if we can find a solution that works for your child, or we may decide that they cannot participate in the study because of this issue.

If your child is in Group 1, 2 or 3, they may be able to participate in prize drawings; they may feel pressure to come to the hospital and take their medications or feel stress when they do not win a prize because they were not eligible for the drawing. Similarly, they may feel upset because of things discussed in the questionnaire.

We may also refer them to one of the many counselors on staff at the Mildmay hospital if warranted and/or desired by your child. The counselor will follow a protocol that includes assessment of severity of the ideation, intent and means for carrying out any intent for suicide, and an appropriate plan of action (e.g., scheduling follow-up counseling, inpatient admission for psychiatric care).

**POTENTIAL BENEFITS TO PARTICIPANT AND THE SOCIETY**

This study helps us better understand how clients at the Mildmay Hospital are taking their medication which will allow us to develop the interventions to better support youth adherence levels.

**COSTS AND COMPESTAION FOR PARTICIPATION**

There are no costs to your child for participating in the study itself. The costs related to ART and procedures that are part of their usual Mildmay clinical care are their responsibility just as they were before they joined the study.

If your child is assigned to Group 1, 2 or 3 and if they achieve the pre-determined adherence level target, **at approximately every** **3 months within the first 24 months of the study period**, they will be invited to participate in a prize drawing where they can win between 500 and 10,000 Ugandan Shillings airtime; the value of the prize is not guaranteed as the prize is drawn by chance.

In addition, if your child either achieves 90% adherence by the end of year 1, or they achieve viral suppression by the end of year 2, or both, they will be entered into an end of year prize draw where they may win up to an amount of 20,000 Ugandan Shillings each time.

We will also compensate your child 30,000 UGX after completing the periodic adherence surveys at months 6, 12, 18 and 24.

**CONFIDENTIALITY**

Your child’s answers, including medical records accessed as part of the study will be kept private. Instead of their name, we will use a number to identify them. All study documents will be kept in a locked office at the Mildmay Hospital for data entry. Computer files will be protected with a password.

We understand that disclosure of your child’s HIV status could be embarrassing or could cause damage to their reputation. Therefore, only researchers will be able to see their answers. We will not share any information your child gives us, and will not share their adherence levels with their doctor; this information will only be available to the research team and will be used for research purposes only. Their name will not be used in any reports or articles we publish. We will not tell their family, their doctors, or anyone else outside the research team what they say or do during the study. However, it is possible that their privacy will be broken. We will do everything we can to prevent this. When we talk about the study in reports or articles, we will hide their name and other things about who they are. All information that identifies them will be destroyed at the end of the study. The data we collect will be stripped of any information that may identify your child or the clinic, and made publicly available at the end of the study.

**VOLUNTARY PARTICIPATION AND OPTION TO WITHDRAWAL**

Participation in this study is your child ‘s choice. If they do not want to be in the study, they can stop taking part at any time. Deciding to stop will not hurt your child. They will still get the same medical care if they decide not to take part in the study.

**QUESTIONS RELATED TO THE STUDY**

If you do not understand something, or if you want more information, please ask now. If you have any questions or concerns about the research, please contact Dr. Barbara Mukasa, Site Principal Investigator, at 0772700816 or Mr. Peter Wabukala, the Study Coordinator, at 0785823296

**RIGHTS OF RESEARCH PARTICIPANTS**

If you have questions about your rights as a research participant, please contact Semei Christopher Mukama, Secretary of the MUREC, at 0392174236 or Uganda National Council for Science and Technology at +256 414 705500/13

**DISSEMINATION OF STUDY FINDINGS**

Your child shall receive communication from the study team about the progress and findings of the study during and/or after the study findings are available to the public. We will not share their name or their client identification number in these communications.

**WRITTEN CONSENT OF THE GUARDIAN OF THE RESEARCH PARTICIPANT**

“I have read (or someone has read to me) the information provided above. I have been given an opportunity to ask questions, and all of my questions have been answered to my satisfaction. I have been given a copy of this form.”

Guardian/ parent has been given a copy of the form YES NO

I consent for my child to participate in the research as described above.

YES (If YES, Continue with signing the consent form). NO

Name of Child __________________________________________

Name of Parent or Guardian ____________________________________

Signature / thumbprint of Parent or Guardian___________________________

Date __________________________________________________

Name of Witness (if unable to read and write) _____________________________________

Signature of Witness ________________________________________

Date ____________________________________________

**SIGNATURE OF STUDY INTERVIEWER**

I have explained the research to the participant and answered all of his/her questions. I believe that he/she understands the information described in this document and freely consents to participate.

Name of Study Interviewer ______________________________

Signature of Study Interviewer ______________________________

Date ______________________________________

### **2.6 Study 2 Non-minor Consent form**

**Consent form for Non-minors Study 2**

**“Goals for Adherence with Low-cost Incentives (GOALS)”**

**Lead Principal Investigator:**

Dr. Sebastian Linnemayr, PhD

Institution of affiliation: RAND Corporation

Address: 1776 Main Street, Santa Monica, CA 90407-2138, US

Phone contact: +1 310 393 0411 ext 6734

Email: slinnema@rand.org

**Principal Investigator:**

Dr. Crystal Haijing Huang, PhD

Institution of affiliation: IDinsight

Address: 44 Tehama Street, San Francisco, CA 94105, US

Phone contact: +1 202 436 1411

Email: crystal.huang@idinsight.org

**Site Principal investigator:**

Dr. Barbara Mukasa

Institution of affiliation: Mildmay Uganda

Address: P.O. Box 24985, Kampala; Uganda

Phone contact: +256 312 210 200 or +256 772 700 816

Email: barbara.mukasa@mildmay.or.ug

**Sponsor:** National Institute of Child Health and Human Development (NICHD), USA

**Collaborating Institutions:** IDinsight, The RAND Corporation, Mildmay Uganda

**INTRODUCTION**

Hello, my name is [insert name of the Study Coordinator]. I work at Mildmay Uganda that in collaboration with the RAND Corporation, and IDinsight, is conducting a study at Mildmay Uganda. The purpose of this study is to understand antiretroviral therapy (ART) medication adherence among young clients at Mildmay Hospital. This study has been approved by an accredited Ugandan-based Research Ethics Committee, the Mildmay Uganda Research Ethics Committee

We would like to thank you for participating in the previous study which measured your adherence using a Wisepill device. We would like to invite you to participate in a new study, which aims to help clients such as yourself take their HIV medication. Before you decide whether or not you want to participate in this new study, we would like to explain why we are doing it, how it may help you, any risks to you, and what is expected of you. After I talk about the details of the study with you, I will ask you if you want to be part of the study and if yes, you will be asked to sign this Informed Consent form.

**STUDY PURPOSE**

Many young people living with HIV have trouble taking their HIV medication every day to receive the most benefit. Therefore, we developed this study called “**Go**als for **A**dherence with **L**ow-cost Incentive**s**” (GOALS), which can help participants such as yourself improve your motivation for taking ART medication, and ultimately reach and maintain a high adherence level. If participants maintain a certain level of adherence, they will be eligible to win prizes between 500 and 10,000 Ugandan Shillings of mobile airtime approximately every 3 months during the first 24 months of the study. Adherence will be measured with a Wisepill device. Additionally, participants may be able to win a larger prize at the end of 12 and 24 months, worth up to 20,000 Ugandan Shillings airtime each.

**STUDY PROCEDURES**

If you decide to participate, you will continue to use the same Wisepill device. You will also be requested to complete a 30-minute survey today if you decide to participate. This questionnaire will be repeated every six months and will include questions about your background, medical history, symptoms, and your attitude to pill taking. Every time you come for a scheduled hospital visit, we will also ask you to come to this research office. Whenever possible, the study activities will be scheduled on days on which you have a visit already scheduled at Mildmay hospital so you don’t have to come to the hospital for study-related reasons only.

A total of 628 HIV positive participants from the Mildmay Hospital who have been receiving ART treatment for at least three months prior to the intervention will take part in the study. You will be assigned to one of the following four groups and will have an equal (i.e., one in four) chance of being in either of the four groups as the assignment is randomly decided by a computer. You will not be able to choose the group you want to be in.

**Participants in Group 1:** For a Group 1 participant **the study coordinator will choose a target based on your current adherence and gradually increase the target every 3 months**. The goal is to achieve a 90% level of adherence by the end of year one. For example, if your initial adherence level is 70%, your adherence target to be eligible to win a prize after 3 months may be 75%. This will continue until you reach 90%.

**Participants in Group 2:** Group 2 participants **can choose their own adherence goal subject to a minimum threshold every 3 months.** The goal is to achieve a 90% level of adherence by the end of year one. For example, if your initial adherence level is 70%, **you** yourself may set your target to be 75%. This will continue until you reach 90%.

**Participants in Group 3:** For Group 3 participants to be eligible for a prize drawing, **you must reach a target adherence level of 90% every 3 months.**

Every 3 months, we will check your adherence and conduct a prize drawing if you reached your target. If you have a hospital visit scheduled when you are due for a prize drawing (every 3 months), we ask that you come to this research office. We will do the prize drawing in person if you reached your target. If you do not have a hospital visit scheduled when you are due for a prize drawing, we will send you an SMS message notifying you of your adherence performance and whether you have achieved your target. If you reached the target, the computer will determine the amount you won; we will inform you of the amount and send you your prize in the form of mobile airtime. If you are in Group 2, we will ask you to text us what you would like your next target to be. All participants will receive a weekly text message with a motivational message.

No personal, private or sensitive information will be included in any text message you receive from us. The text message will not include words such as ‘HIV’ or ‘ART medication’, and will not share or imply your HIV status. For example, a text message could say *“Hello, this is GOALS. Congratulations! You are at 70% and your target was 85%. The computer determined that your prize is 10,000 UGX. We will now send you this as Airtime”.*

For the first 12 months, participants in Groups 1, 2, and 3 can win a prize between 500 and 10,000 Ugandan Shillings in mobile airtime every 3 months if they reach the pre-determined target.

After 12 months, if you achieve 90% adherence, we will ask you to maintain this level in order to be eligible for a prize drawing approximately every 3 months. If you do not achieve 90% adherence, you will be given another 6 months to improve to 90%. There will be a larger prize drawing if you achieve 90% adherence at the end of month 12 or month 18, depending on the category of adherence you fall under. You will be entered into a prize draw where you may win up to 20,000 Ugandan Shillings of airtime.

Similarly, a larger prize draw will happen if you achieve viral suppression by the end of month 24 or month 30, depending on the category of adherence you fall under after month 12.

After these 30 months, there will be no more prize draws but we will keep measuring your adherence for another 6 months to see how you take your medication during this time.

**Participants in Group 4:** **You will receive the usual standard of care** as offered to all clients at Mildmay Hospital. You will also receive weekly text messages, but there will not be prize drawings in this group. We will ask you to use your Wisepill device for 36 months and come to the research office each time you come to Mildmay for a hospital visit. We will also give you 30,000 UGX for transport on the 6^th^, 12^th^, 18^th^ and 24^th^ month when you come back to complete adherence surveys.

I can’t tell you which group you will be in until you tell me whether you agree to continue in the study.

**SCHEDULE OF FOLLOW UP VISITS**

No matter which group you are assigned to, you will continue to come to Mildmay Hospital for your HIV care on your regularly scheduled days. If you decide to stop taking your ART medication before reaching the end of the study, you will be asked to still come to the hospital for all the remaining study visits. If you wish to leave the study and not continue with the scheduled study visits, you are free to do so.

# USE OF ELECTRONIC DEVICE TO MEASURE ADHERENCE

Over the course of the study, you will be asked to use the Wisepill device and bring it to each hospital visit. Please note that at any point, you may choose not to continue participation in the study. You should not participate in the study if you do not want to or cannot use the Wisepill device. In intervention Groups 1, 2 and 3, the prize drawings which happen approximately every 3 months are based on achieving your specific adherence level measured by the Wisepill device.

**Risk AND DISCOMFORTS**

Being in this study may involve some risks and discomforts. Participating in the study requires participants to use the electronic Wisepill device. You will be asked to only remove the ART medication from the device when you are about to swallow the medication. If you don’t take the pills the way the doctor tells you to, this may reduce the effectiveness of the medication and increase the risk of developing drug resistance. If using the electronic device to take your medication poses a big problem for you, you can stop using it. You may not feel comfortable using the device if, for example, people who don’t know your HIV status ask you what it is; in such cases, we have to discuss if we can find a solution that works for you, or we may decide that you cannot participate in the study because of this issue.

If you are in Group 1, 2 or 3, you may be able to participate in prize drawings; you may feel pressure to come to the hospital and take your medications or feel stress when you do not win a prize because you were not eligible for the drawing. Similarly, you may feel upset because of things discussed in the questionnaire.

We may also refer you to one of the many counselors on staff at the Mildmay hospital if warranted and/or desired by yourself. The counselor will follow a protocol that includes assessment of severity of the ideation, intent and means for carrying out any intent for suicide, and an appropriate plan of action (e.g., scheduling follow-up counseling, inpatient admission for psychiatric care).

**POTENTIAL BENEFITS TO PARTICIPANT AND THE SOCIETY**

The study helps us better understand how clients at the Mildmay Hospital are taking their medication which will allow us to develop the interventions to better support youth adherence levels.

**COSTS AND COMPESTAION FOR PARTICIPATION**

There are no costs to you for participating in the study itself. The costs related to ART and procedures that are part of your usual Mildmay clinical care are your responsibility just as they were before you joined the study.

If you are assigned to Group 1, 2 or 3 and if you achieve the pre-determined target adherence level **approximately every** **3 months within the first 24 months of the study period**, you will be invited to participate in a prize drawing where you can win between 500 and 10,000 Ugandan Shillings airtime; the value of the prize is not guaranteed as the prize is drawn by chance.

In addition, if you either achieve 90% adherence by the end of year 1, or you achieve viral suppression by the end of year 2, or both, you will be entered into an end of year prize draw where you may win up to an amount of 20,000 Ugandan Shillings each time.

We will also compensate you 30,000 UGX after completing the periodic adherence surveys at months 6, 12, 18 and 24.

**CONFIDENTIALITY**

Your answers, including medical records accessed as part of the study will be kept private. Instead of your name, we will use a number to identify you. All study documents will be kept in a locked office at the Mildmay Hospital for data entry. Computer files will be protected with a password.

We understand that disclosure of your HIV status could be embarrassing or could cause damage to your reputation. Therefore, only researchers will be able to see your answers. We will not share any information you give us, and will not share your adherence levels with your doctor; this information will only be available to the research team and will be used for research purposes only. Your name will not be used in any reports or

articles we publish. We will not tell your family, your doctors, or anyone else outside the research team what you say or do during the study. However, it is possible that your privacy will be broken. We will do everything we can to prevent this. When we talk about the study in reports or articles, we will hide your name and other things about who you are. All information that identifies you will be destroyed at the end of the study. The data we collect will be stripped of any information that may identify you or the Hospital, and made publicly available at the end of the study.

**VOLUNTARY PARTICIPATION AND OPTION TO WITHDRAWAL**

Participation in this study is your choice. If you do not want to be in the study, you can stop taking part at any time. Deciding to stop will not hurt you. You will still get the same medical care if you decide not to take part in the study.

**QUESTIONS RELATED TO THE STUDY**

If you do not understand something, or if you want more information, please ask now. If you have any questions or concerns about the research, please contact Dr. Barbara Mukasa, Site Principal Investigator, at 0772700816 or Mr. Peter Wabukala, the Study Coordinator, at 0785823296

**RIGHTS OF RESEARCH PARTICIPANTS**

If you have questions about your rights as a research participant, please contact Semei Christopher Mukama, Secretary of the MUREC, at 0392174236 or Uganda National Council for Science and Technology at +256 414 705500/13

**DISSEMINATION OF STUDY FINDINGS**

You shall receive communication from the study team about the progress and findings of the study during and/or after the study findings are available to the public. We will not share your name or your client identification number in these communications.

**WRITTEN CONSENT OF RESEARCH PARTICIPANT**

“I have read (or someone has read to me) the information provided above. I have been given an opportunity to ask questions, and all of my questions have been answered to my satisfaction. I have been given a copy of this form.”

I consent to participate in the research as described above.

I have chosen to take a copy of this consent form

YES (If YES, Continue to consenting). NO

YES (If YES, Continue with signing the consent form). NO

Name of Participant ________________________________________

Signature / thumbprint of Participant _______________________________________

Date __________________________________________________________

Name of Witness (if unable to read and write) _______________________________________

Signature / of Witness ____________________________________

Date ___________________________________________________

**SIGNATURE OF INVESTIGATOR/STUDY INTERVIEWER**

I have explained the research to the participant and answered all of his/her questions. I believe that he/she understands the information described in this document and freely consents to participate.

Name of Study Interviewer ____________________________________________

Signature of Study Interviewer __________________________________________

Date _________________________________________________
